# A prognostic model based on clusters of molecules related to epithelial–mesenchymal transition for idiopathic pulmonary fibrosis

**DOI:** 10.3389/fgene.2022.1109903

**Published:** 2023-01-06

**Authors:** Jiarui Zhao, Can Wang, Rui Fan, Xiangyang Liu, Wei Zhang

**Affiliations:** ^1^ College of Traditional Chinese Medicine, Shandong University of Traditional Chinese Medicine, Jinan, Shandong, China; ^2^ College of First Clinical Medicine, Shandong University of Traditional Chinese Medicine, Jinan, China

**Keywords:** idiopathic pulmonary fibrosis, prognostic model, epithelial-mesenchymal transition, bioinformatics, bronchoalveolar lavage cells

## Abstract

**Background:** Most patients with idiopathic pulmonary fibrosis (IPF) have poor prognosis; Effective predictive models for these patients are currently lacking. Epithelial–mesenchymal transition (EMT) often occurs during idiopathic pulmonary fibrosis development, and is closely related to multiple pathways and biological processes. It is thus necessary for clinicians to find prognostic biomarkers with high accuracy and specificity from the perspective of Epithelial–mesenchymal transition.

**Methods:** Data were obtained from the Gene Expression Omnibus database. Using consensus clustering, patients were grouped based on Epithelial–mesenchymal transition-related genes. Next, functional enrichment analysis was performed on the results of consensus clustering using gene set variation analysis. The gene modules associated with Epithelial–mesenchymal transition were obtained through weighted gene co-expression network analysis. Prognosis-related genes were screened *via* least absolute shrinkage and selection operator (LASSO) regression analysis. The model was then evaluated and validated using survival analysis and time-dependent receiver operating characteristic (ROC) analysis.

**Results:** A total of 239 Epithelial–mesenchymal transition-related genes were obtained from patients with idiopathic pulmonary fibrosis. Six genes with strong prognostic associations (C-X-C chemokine receptor type 7 [*CXCR7*], heparan sulfate-glucosamine 3-sulfotransferase 1 [*HS3ST1*], matrix metallopeptidase 25 [*MMP25*], murine retrovirus integration site 1 [*MRVI1*], transmembrane four L6 family member 1 [*TM4SF1*], and tyrosylprotein sulfotransferase 1 [*TPST1*]) were identified *via* least absolute shrinkage and selection operator and Cox regression analyses. A prognostic model was then constructed based on the selected genes. Survival analysis showed that patients with high-risk scores had worse prognosis based on the training set [hazard ratio (HR) = 7.31, *p* < .001] and validation set (HR = 2.85, *p* = .017). The time-dependent receiver operating characteristic analysis showed that the area under the curve (AUC) values in the training set were .872, .905, and .868 for 1-, 2-, and 3-year overall survival rates, respectively. Moreover, the area under the curve values in the validation set were .814, .814, and .808 for 1-, 2-, and 3-year overall survival rates, respectively.

**Conclusion:** The independent prognostic model constructed from six Epithelial–mesenchymal transition-related genes provides bioinformatics guidance to identify additional prognostic markers for idiopathic pulmonary fibrosis in the future.

## 1 Introduction

Idiopathic pulmonary fibrosis (IPF) is an interstitial lung disease; Its causes are unknown but may be associated with genetic, environmental, and occupational exposure (Taskar and Coultas, 2006; Park et al., 2021). The clinical presentation of IPF includes dyspnea and an irritating dry cough, among other symptoms (Raghu et al., 2011). Although the incidence of IPF is only approximately .09–1.30 per 10,000 people worldwide (Maher et al., 2021), its risk is increasing annually (Richeldi et al., 2017). There are many limitations to IPF treatment in current clinical practice. Pirfenidone and nintedanib are the main therapeutic agents and improve patient quality of life and clinical symptoms. However, both are associated with adverse effects, such as thrombocytopenia and gastrointestinal discomfort, and neither is effective in improving lung function (Spagnolo et al., 2021). Further, some patients experience slow disease progression, but other patient progress rapidly toward death (Lederer and Martinez, 2018). At present, a clinical method to determine the prognosis of IPF is lacking, and thus, it is necessary to screen for IPF prognosis-related biomarkers to further advance diagnostics and precision medicine.

Epithelial–mesenchymal transition (EMT) leads to the loss of contact adhesion and apical–basal polarity in epithelial cells based on a change in gene regulation, which changes the cytoskeletal and mesenchymal features of the extracellular matrix (Lamouille et al., 2014; Dongre and Weinberg, 2019). Many extracellular ligands, such as epidermal growth factor, interleukin-1, and Wnt, bind to surface receptors during EMT and activate multiple transcription factors through multiple pathways, leading to decreased expression of adhesion molecules (Lin and Wu, 2020; Jayachandran et al., 2021). EMT is a physiological process that occurs during embryonic development. EMT is also a pathological process that occurs in many diseases (Mittal, 2018), such as breast cancer (Scimeca et al., 2021) and lung cancer (Mittal, 2016), among others. Studies have shown that the development of fibroblastic foci in IPF is closely related to the EMT (DeMaio et al., 2012; Yamaguchi et al., 2017). The mechanisms underlying EMT in IPF are mesenchymal cell abnormalities and extracellular matrix remodeling, ultimately causing abnormal activation of repair pathways in the damaged alveolar epithelium (Hewlett et al., 2018). The EMT process in IPF is influenced by multiple pathways and biological processes, so it more likely to obtain a better prognostic model based on the EMT process. Prognosis-related study is also an attempt to further explore the specific mechanisms of the EMT process in IPF.

At present, with the development of microarray and sequencing technology, genetic testing technology is becoming increasingly common. Based on bioinformatics approach, one study explored a prognostic model for lung adenocarcinoma from the perspective of pyroptosis-related factors (Lin et al., 2022), and one study explored a prognostic model for IPF from the perspective of immune-related chromatin regulatory genes (Li et al., 2022). However, translating the clinical and prognostic value of EMT-related genes to IPF requires extensive research. Thus, it is necessary to screen prognosis-related genes for IPF at the molecular level, based on EMT processes, and then construct prognostic models for clinical purposes.

Bronchoalveolar lavage (BAL) is the subject of a common ancillary test for IPF diagnosis (Meyer et al., 2012; Patel et al., 2021). Since bronchoalveolar lavage fluid (BALF) better reflects the exudation of inflammatory factors and mediators in IPF and improves the accuracy of IPF biomarker construction, BAL cell samples were selected for both the training and validation sets of this study (Xia et al., 2021; Wang et al., 2022). First, differential EMT-related genes were identified in patients with IPF *via* consensus clustering and weighted co-expression network analysis (WGCNA). Additionally, an enrichment analysis for EMT-associated genes was performed, and then, genes associated with IPF prognosis were filtered through least absolute shrinkage and selection operator (LASSO) and Cox regression analyses. Through the construction and validation of this prognostic model, new evidence is provided that will be helpful in clinical situations and in determining the prognostic outcomes of patients with IPF.

## 2 Materials and methods

### 2.1 Dataset acquisition and organization

**FIGURE 1:**
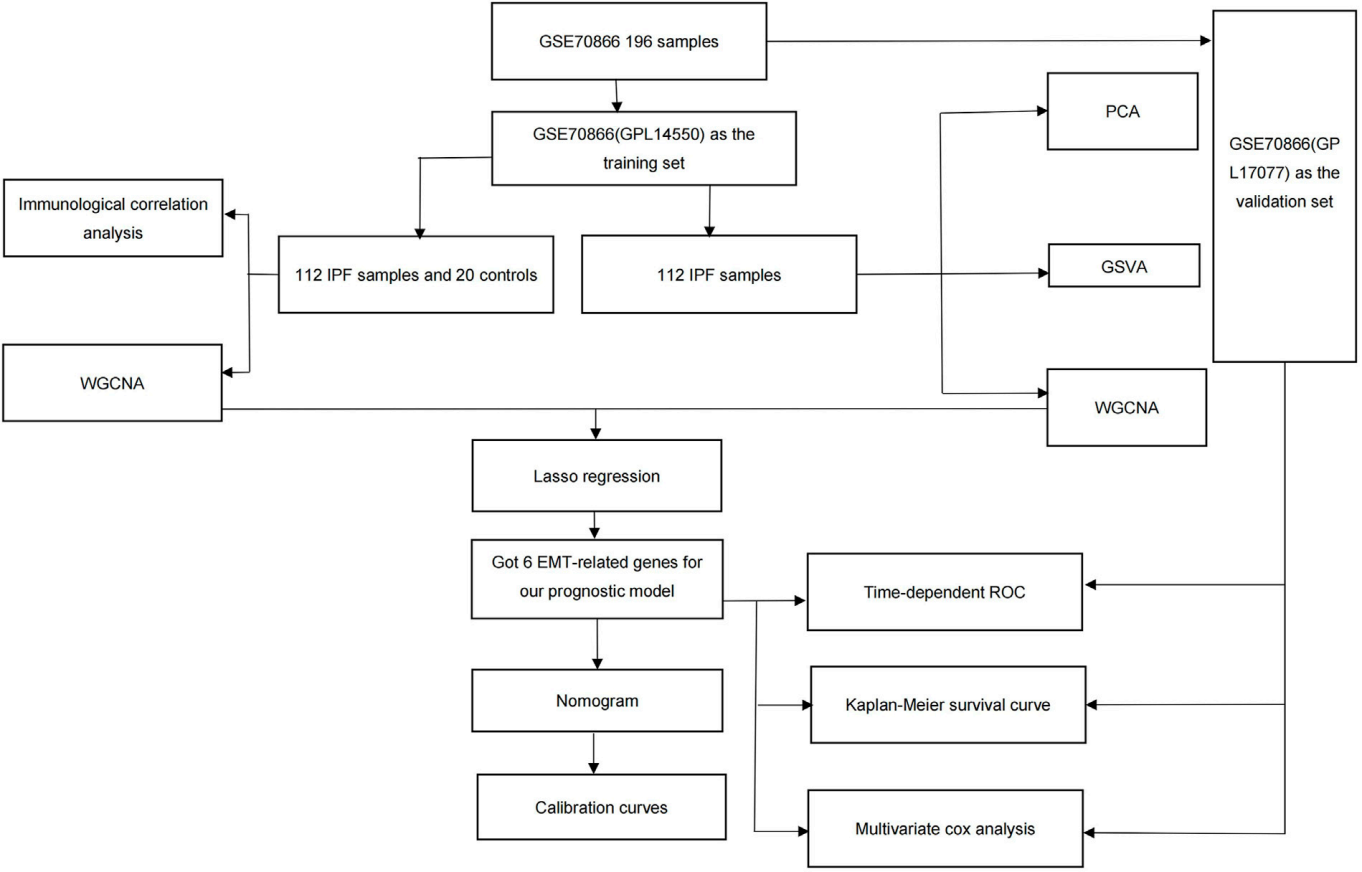
Study outline.

The original data were obtained from the Gene Expression Omnibus (GEO) database (https://www.ncbi.nlm.nih.gov/geo/), using the criteria “idiopathic interstitial lung fibrosis,” “sample size greater than 100,” “including clinical information,” and “expression profiling by array.” The dataset GSE70866 was downloaded for this study using the “GEOquery” R package (Davis and Meltzer, 2007). These data consisted of mRNA expression of 196 BAL cell samples from three independent cohorts and two platforms (Prasse et al., 2019). Depending on different platforms, the Freiburg, Germany (62 patients and 20 healthy donors) and Siena, Italy (50 patients) cohorts (GPL14550) were used as training sets, whereas the Leuven, Belgium (64 patients) cohort (GPL17077) was used as the validation set. The quality of the raw data was evaluated using the PCA method. EMT-related genes for reference were obtained from the HALLMARK EPITHELIAL MESENCHYMAL TRANSITION gene set in the Molecular Signatures Database (MSigDB) (Liberzon et al., 2015). This study was not required to undergo ethical review because all data were sourced from open-source databases; the detailed process is shown in Figure 1.

### 2.2 Acquisition of EMT-Related genes

The original data were corrected and normalized using the “limma” R package (Smyth, 2005). Differences between control samples and samples of patients with IPF were analyzed using the training set. Here, 110 differentially expressed genes (DEGs) were obtained using a Benjamini–Hochberg-adjusted *p*-value less than .05 and an absolute fold-change value (log_2_FC) greater than 1.5. The intersection between the 110 DEGs and the EMT-related genes from the MSigDB was determined, and from this, four genes were obtained. A circle map for these four genes was then generated using the “RCircos” R package (Zhang et al., 2013).

### 2.3 Immunological correlation analysis

Immune cell infiltration in all samples was calculated using the CIBERSORT algorithm and LM 22 signature matrix (Newman et al., 2015). The CIBERSORT algorithm has a total ratio of one for 22 immune cell types in one sample. The expression differences associated with 22 immune cell types between control and IPF groups were compared using the “reshape2” and “ggpubr” R packages. The Spearman correlation coefficients between the four EMT-related genes and immune cell infiltration were plotted using the “ggplot two” R package.

### 2.4 Consensus clustering and principal component analysis (PCA)

Consensus clustering analysis was performed on the training set of IPF samples based on the four EMT-related genes using the “ConsensusClusterPlus” R package (Wilkerson and Hayes, 2010). The 112 IPF samples were classified into different categories using 1,000 calculations. Based on the results of the consensus score, cumulative distribution function (CDF), and area under the CDF, clusters 1 and 2 were obtained based on the best K (K = 2) value for the clustering effect.

### 2.5 Enrichment analysis

The consensus clustering results were analyzed using the “GSVA” package (Hänzelmann et al., 2013). Gene files from the Gene Ontology (GO) (c5.go.symbols.gmt) and Kyoto Encyclopedia of Genes and Genomes (KEGG) (c2.cp.kegg.symbols.gmt) databases, which were obtained from the MSigDB [24], were analyzed, and enrichment results for 112 samples were obtained in terms of pathways and biological functions. The most distinct pathways and biological functions in cluster 1 and 2 were selected from their functional enrichment levels using the “limma” package (Smyth, 2005).

### 2.6 WGCNA

WGCNA was performed using the WGCNA package (Langfelder and Horvath, 2008) for the top 15% of mutated genes in all 132 samples (divided into control and IPF samples) and 112 IPF samples (divided into cluster 1 and cluster 2). All modules were restricted to be greater than 100, and the best soft thresholding power, as well as the topological overlap matrix (TOM) and TOM dissimilarity measure (1-TOM), were obtained based on an adjacency matrix. Different colors were randomly assigned to the co-expressed gene modules, and the most significantly different modules were selected for further analysis.

### 2.7 LASSO and cox regression analyses

The intersection between the two modules with the most significant *p*-values in the WGCNA of the 132 samples and the 112 IPF samples was determined, and 239 intersecting genes were obtained. LASSO (“glmnet” R package) and Cox regression analyses were performed to select EMT-related prognostic genes to form the prognosis model. Based on the LASSO regression, we obtained the EMT-related prognostic genes and their corresponding coefficients. We multiplied the gene expressions with the corresponding coefficients and summed all of them (Wang et al., 2022). The risk score formula was constructed as follows:

$$\begin{array}{ll} Risk\;score & =corresponding\;coefficient\;of\;gene\;1\times expression\;of\;gene\;1+corresponding\;coefficient\;of\;gene\;2\times expression\;of\;gene\;2\\ & +corresponding\;coefficient\;of\;gene\;3\times expression\;of\;gene\;3+\cdots +corresponding\;coefficient\;of\;gene\;n\times expression\;of\;gene\;n \end{array}$$

This formula was used to calculate the risk scores for patients with IPF.

### 2.8 Model construction and evaluation

The prognostic nomogram and calibration curves for 1-, 2-, and 3-year overall survival rates were plotted using the “rms” R package. The “timeROC” and “survminer” R packages were used to create time-dependent ROC and survival analysis plots, respectively. In the training set, there were 19 females and 93 males, and the average age of all patients was 67.179 years old (Supplementary Table S1). In the validation set, there were 13 females and 51 males, and the average age of all patients was 68.250 years old (Supplementary Table S2). The model was tested based on a multifactorial Cox analysis with age and sex, and the Leuven, Belgium (64 patients) cohort was used to test the model.

### 2.9 Statistical analysis and graphing

Statistical analysis and graphical plotting were performed using R 4.1.2. The Shapiro–Wilk test was used for the normal distribution of continuous variables, and the Bartlett’s test was used for variance chi-square analysis. The log-rank test was used for survival analysis. When the data met the requirements of variance chi-square and normal distribution, an independent samples *t*-test or Wilcoxon signed rank test was used for analysis. If the Pearson’s correlation coefficient was greater than .6, it was considered that there was a correlation. If the *p*-value was less than .05, it was considered significant.

## 3 Results

### 3.1 Significantly changed EMT-Related genes in IPF

**FIGURE 2:**
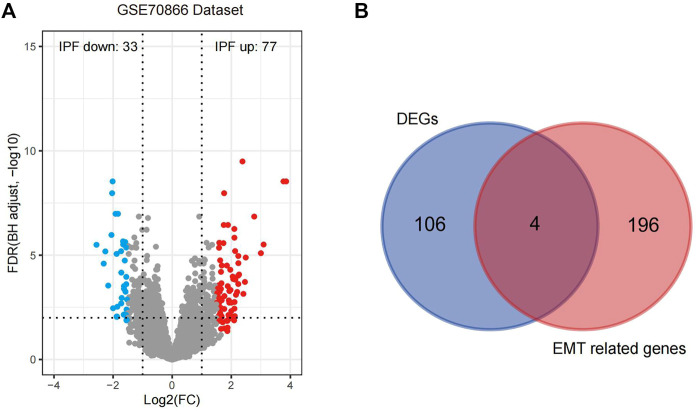
Acquisition and analysis of four EMT-related DEGs in IPF. **(A)** The 110 DEGs identified are displayed in the volcano plot based on the criteria of *p* < .05 and log_2_FC > 1.5. **(B)** The EMT-related genes are presented in a Venn diagram. IPF, idiopathic pulmonary fibrosis; EMT, epithelial–mesenchymal transition; DEGs, differentially expressed genes.

By evaluating the quality of the raw data, we can see that the outlier samples were little and the data can be further analyzed and processed (Supplementary Figure S1A). The 20 controls and 112 IPF samples from GSE70866 (GPL14550) were tested for differential analysis based on a Benjamini–Hochberg-adjusted *p*-value less than .05 and an absolute log_2_FC value greater than 1.5. A total of 77 significantly upregulated DEGs and 33 significantly downregulated DEGs were identified; those were displayed using a volcano plot (Figure 2A; Supplementary Table S3). Since IPF is closely related to EMT-related processes, the intersection between the 110 DEGs and 200 EMT-related genes from the MSigDB was determined (Supplementary Table S4), and four related genes were obtained, namely, secreted phosphoprotein 1 (*SPP1*), integrin beta-3 (*ITGB3*), high temperature requirement 1 (*HTRA1*), and tissue inhibitor of metalloproteinase 3 (*TIMP3*), which were significantly altered in IPF; these were plotted using a Venn diagram (Figure 2B). The specific locations of these four genes on the chromosome were determined based on mapping using a gene circle (Supplementary Figure S1B). To explore potential interactions among these four genes, the correlations between them were calculated (in Supplementary Figure S1C). Only positive correlations were identified, and the strongest correlation was observed between *HTRA1* and *SPP1* (correlation = .75).

### 3.2 Immune cell infiltration analysis

**FIGURE 3:**
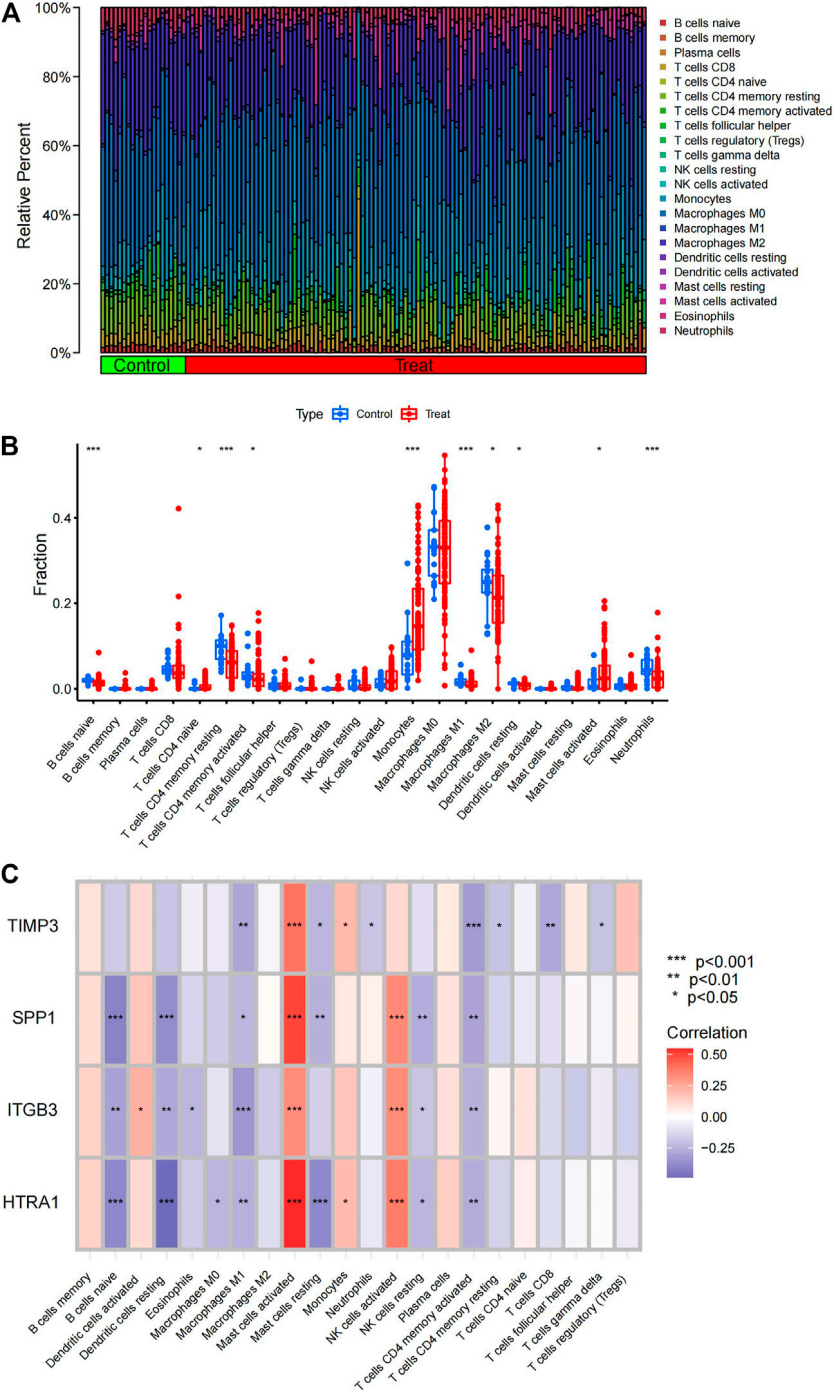
Analysis of immune cell type infiltration. **(A)** Relative abundance of immune cell types in the IPF and control samples. **(B)** Differences in immune cell infiltration between IPF and control samples. **(C)** EMT-related DEGs displayed based on an immune correlation analysis. IPF, idiopathic pulmonary fibrosis; EMT, epithelial–mesenchymal transition; DEGs, differentially expressed genes.

Many immune cell types are expressed abnormally in the development of IPF, and EMT process is also inextricably linked to immune responses. Exploring different immune cell types between disease and control samples by immune infiltration analysis, we hope to provide more ideas for subsequent analysis. CIBERSORT scores were obtained using the CIBERSORT algorithm (Supplementary Table S5) and relative abundances were plotted (Figure 3A). Based on the box plots, memory CD4^+^ T cell, M1 macrophage, M2 macrophage, dendritic cell, neutrophil, and naive B cell populations were significantly decreased, whereas naive T cell, monocyte, and mast cell populations were significantly increased, indicating that IPF development might be related to immune cell type imbalances (Figure 3B). The aforementioned four EMT-related DEGs were further subjected to an immune correlation analysis (Figure 3C). These four genes were positively correlated with activated mast cells with *p*-values <.001, suggesting that the increased response to mast cells in IPF might be closely related to the EMT process. The above results suggest that the EMT process in IPF can be further investigated in the perspective of immune abnormalities in the future.

### 3.3 Consensus clustering of IPF samples

**FIGURE 4:**
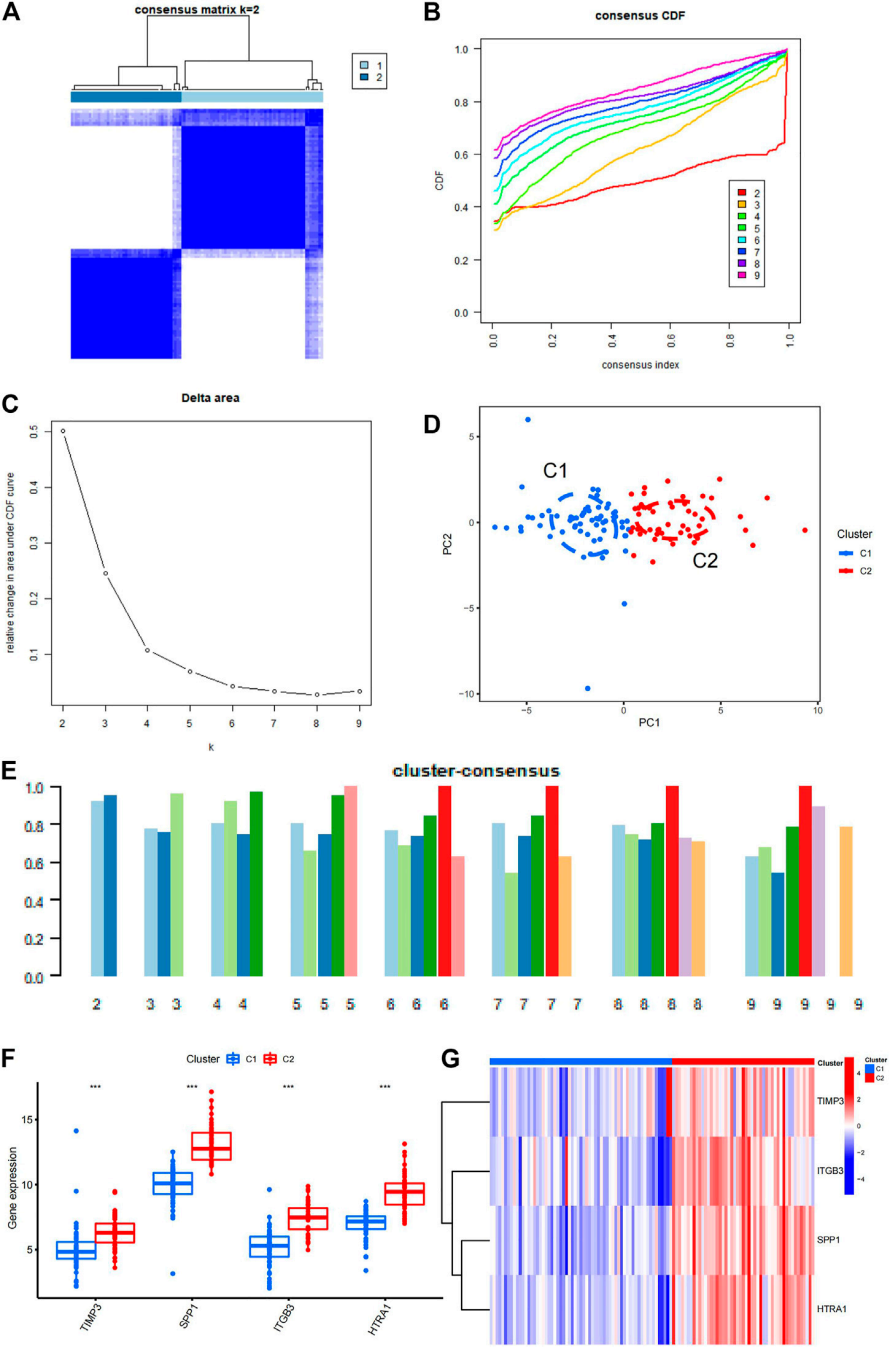
Consensus clustering of IPF samples. **(A)** Consensus clustering matrix constructed based on the final K = 2. **(B)** Consensus CDF. The different color numbers in the figure represent the different K from two to nine. The horizontal coordinate represents consensus index and the vertical coordinate represents CDF value. **(C)** Area under the CDF. The horizontal coordinate represents the different K from two to nine and the vertical coordinate represents the change in area under the CDF curve. **(D)** PCA of the two clusters. C1 is cluster 1, C2 is cluster 2. **(E)** The cluster-consensus plot demonstrates the consensus clustering results. The horizontal coordinate represents the different K from two to nine and the vertical coordinate represents the consistency score. **(F)** The box plot shows the significant differences in the four EMT-related genes between the two clusters. C1 is cluster 1, C2 is cluster 2. **(G)** The heatmap shows the specific differences in the four genes between the two clusters. C1 is cluster 1, C2 is cluster 2. IPF, idiopathic pulmonary fibrosis; CDF, cumulative distribution function; PCA, principal component analysis; EMT, epithelial–mesenchymal transition.

Using four EMT-related genes, a consensus clustering of IPF samples was performed. The aim of this analysis was to group IPF samples by the four EMT-related genes, so we can get more EMT-related genes in next analyses. The samples could be well separated when K = 2, so the clustering effect was considered optimal when K = 2 (Figure 4A). When consensus index varied from .2 to .8, the CDF curve of K = 2 was the most stable one; this supported the choice to divide the IPF samples into two cluster when K = 2 (Figure 4B). When K was changed from two to nine, the area under the CDF curve changed significantly from K = 2 to K = 3 (Figure 4C), and the consistency scores of cluster 1 and cluster 2 were both greater than .9 (Figure 4E), this also supported the choice to divide the IPF samples into two cluster when K = 2. Based on the above analysis, the 112 patients with IPF were divided into cluster 1 (63 samples) and cluster 2 (49 samples) (Supplementary Table S6). To test the clustering effect, PCA was performed on the two clusters, which revealed that the 112 patients with IPF could be divided into two clusters with no outlier samples, suggesting that the clustering was effective (Figure 4D). The box plot demonstrated that the four genes related to EMT were significantly differentially expressed between the two cluster groups (Figure 4F). The heatmap also further reflected the specific expression of the four genes in the two cluster groups (Figure 4G).

### 3.4 Functional enrichment analyses

To provide additional information about the biological function and pathway differences between clusters 1 and 2, gene set variation analysis (GSVA) was performed. Using GSVA, butanoate metabolism, biosynthesis of unsaturated fatty acids, limonene and pinene degradation, propanoate metabolism, and peroxisome were enriched in cluster 2. Primary bile acid biosynthesis and tyrosine metabolism were reduced in cluster 2 (Supplementary Figure S1D). Several GO biological processes such as BBSome, membrane attack complex, positive regulation of calcium ion transmembrane transporter activity, positive regulation of memory T cell differentiation, and cation chloride symporter activity were increased in cluster 2. A few GO biological processes including positive regulation of extracellular exosome assembly were decreased in cluster 2 (Supplementary Figure S1E). Through the above GSVA analysis, we found significant differences in the biological processes between cluster 1 and cluster 2. The results indicated that there were indeed some differences between different subgroups of patients in IPF, so we can continue the WGCNA analysis and prognostic analysis in the following.Together, these data suggested that the EMT process in IPF might be closely related to abnormal metabolic functions in the organism. It provided a direction for us to further investigate the specific mechanism of EMT-related genes in IPF.

### 3.5 Selection of gene module *via* WGCNA

**FIGURE 5:**
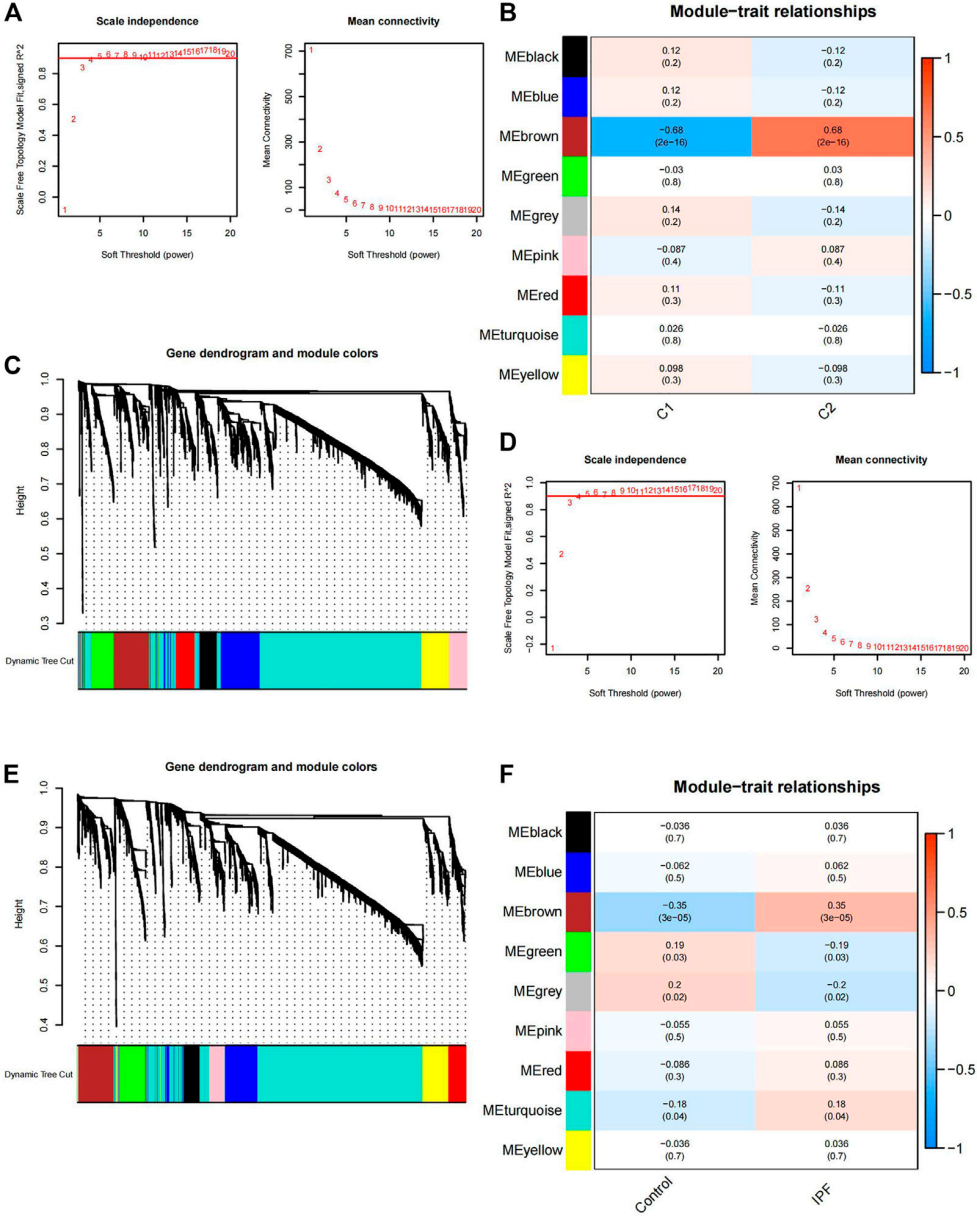
Gene module selection using WGCNA. **(A)** Selection of the soft threshold power in clusters one and two. When the scale-free fit index is .9, the minimum soft threshold is 4. **(B)** Gene clustering tree in cluster 1 and cluster 2. C1 is cluster 1, C2 is cluster 2. **(C)** Correlation heatmap between the co-expression modules in clusters 1 and 2; the brown module has the highest correlation and the lowest *p*-value (*P* = 2e-16) in cluster 1 (correlation = −.68) and cluster 2 (correlation = .68). **(D)** Selection of the soft threshold power for the 112 IPF and 20 control samples. When the scale-free fit index is .9, the minimum soft threshold is 4. **(E)** Gene clustering tree of the 112 IPF and 20 control samples. **(F)** Correlation heatmap between the co-expression modules in the 112 IPF and 20 control samples; the brown module has the highest correlation and the lowest *p*-value (*P* = 3e-05) in cluster 1 (correlation = −.35) and cluster 2 (correlation = .35). WGCNA, weighted gene co-expression network analysis; IPF, idiopathic pulmonary fibrosis.

Using the WGCNA algorithm, clusters 1 and 2 were generated for co-expression network building, and the top 15% of genes showing the highest variance for the calculation were selected. The minimum soft threshold was four when the scale-free fit index was .9 (Figure 5A). The best soft threshold was selected to construct the co-expression network and produce the gene clustering tree (Figure 5B). After clustering similar genes into one category and plotting the correlation heatmap between modules (Figure 5C), the brown module had the highest correlation and lowest *p*-value (*P* = 2e-16) in cluster 1 (correlation = −.68) and cluster 2 (correlation = .68). Thus, 277 genes in the brown module (Supplementary Table S7) were selected for subsequent analysis.

The IPF and control samples were also used for co-expression network building, and the top 15% of genes with the highest variance for the calculation were selected. The scale-free fit index was .9 when the soft threshold was 4 (Figure 5D). The gene clustering tree under the optimal soft threshold conditions (Figure 5E) and the correlation heatmap between similar gene modules were plotted (Figure 5F). The brown module had the highest correlation and the smallest *p*-value (*P* = 3e-05) for the control (correlation = −.35) and IPF samples (correlation = .35). Thus, 271 genes in the brown module (Supplementary Table S8) were selected for subsequent analysis.

### 3.6 Prognostic model associated with EMT

**FIGURE 6:**
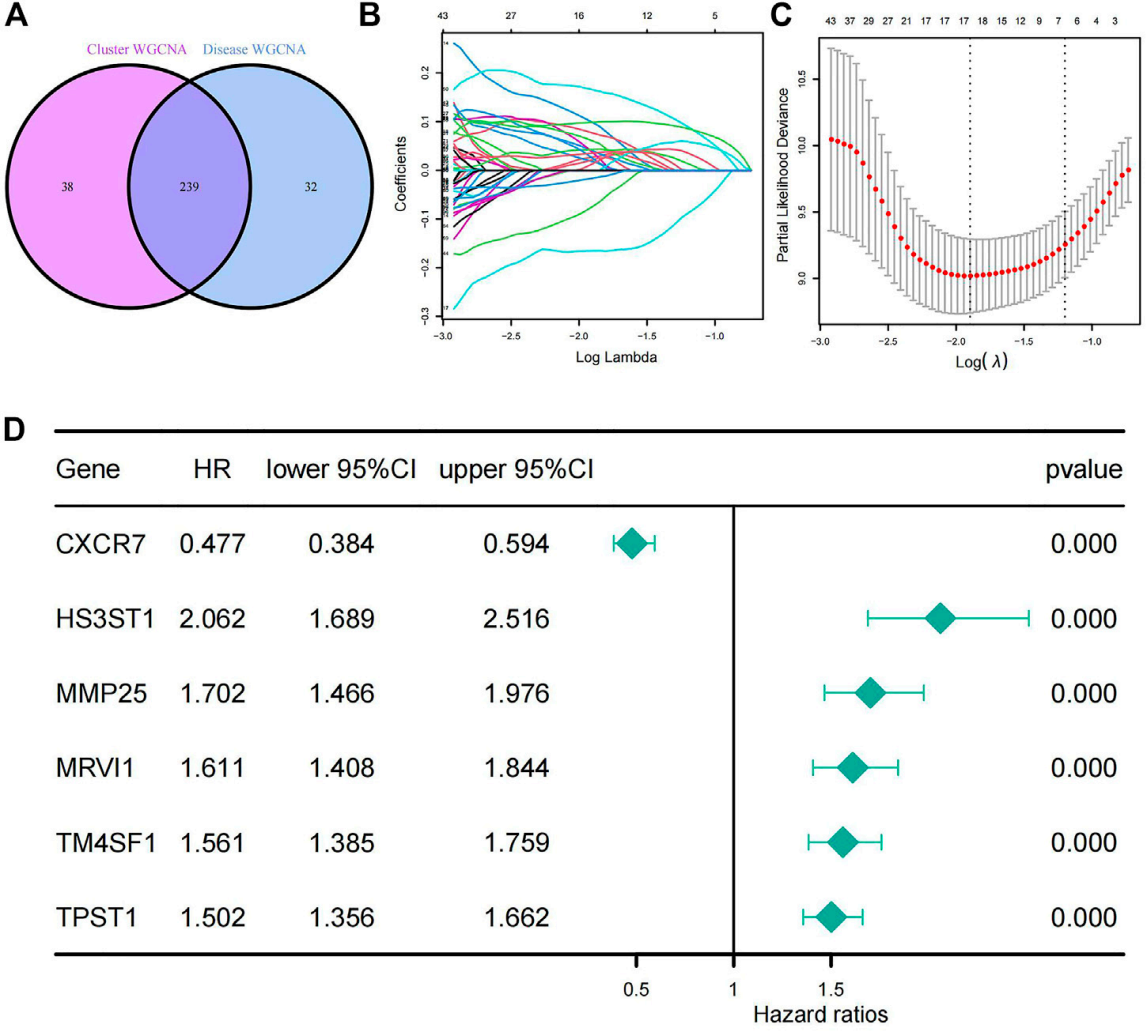
Generation of a prognostic model for patients with IPF. **(A)** The Venn diagram of the 239 EMT-related genes which got from the intersection of WGCNA results. **(B)** LASSO coefficient profiles of the 239 genes. **(C)** The largest λ value (λ = 6) in the mean square error within the standard error. **(D)** Univariate Cox analysis of the six selected genes. All *p*-values from the univariate Cox analysis of the six genes are less than .000. IPF, idiopathic pulmonary fibrosis; LASSO, least absolute shrinkage and selection operator.

The two groups of genes obtained from the above WGCNA analysis were intersected and 239 intersecting genes were obtained (Supplementary Table S9; Figure 6A). Cluster 1 and cluster 2 were clustered using EMT-related genes and the 239 intersecting genes were derived from the subsequent WGCNA analysis, so these 239 genes were related to the EMT process in IPF. We used these 239 genes as EMT-related genes for the filtering and construction of our prognostic model. Through LASSO analysis, six genes (C-X-C chemokine receptor type 7 [*CXCR7*], heparan sulfate-glucosamine 3-sulfotransferase 1 [*HS3ST1*], matrix metallopeptidase 25 [*MMP25*], murine retrovirus integration site 1 [*MRVI1*], transmembrane four L6 family member 1 [*TM4SF1*], and tyrosylprotein sulfotransferase 1 [*TPST1*]) and their corresponding coefficients were acquired (Figure 6B, C; Supplementary Table S10). These genes were further validated *via* univariate Cox analysis. All *p*-values for the six genes were less than .05, suggesting that all six genes were associated with prognosis. The hazard ratio (HR) of *CXCR7* was less than 1 (HR = .477), whereas the HRs of the other five genes—*HS3ST1* (HR = 2.062), *MMP25* (HR = 1.702), *MRVI1* (HR = 1.611), *TM4SF1* (HR = 1.561), and *TPST1* (HR = 1.502)—were all greater than 1. This indicated that, except for *CXCR7*, these genes were positively associated with prognosis (Figure 6D). These six genes were identified as prognosis-related genes and were combined with their corresponding coefficients to construct a prognostic model. The formula for the risk score for this model is as follows:

$$\begin{array}{c} Risk\;score=CXCR7\times \left(-0.0881067640076111\right)\\ +HS3ST1\times \left(0.0892716591390481\right)\\ +MMP25\times \left(0.0138121800572637\right)\\ +MRVI1\times \left(0.0353958603331637\right)\\ +TM4SF1\times \left(0.0585011127027571\right)\\ +TPST1\times \left(0.0853951015444165\right) \end{array}$$

### 3.7 Evaluation and validation of prognostic models

**FIGURE 7:**
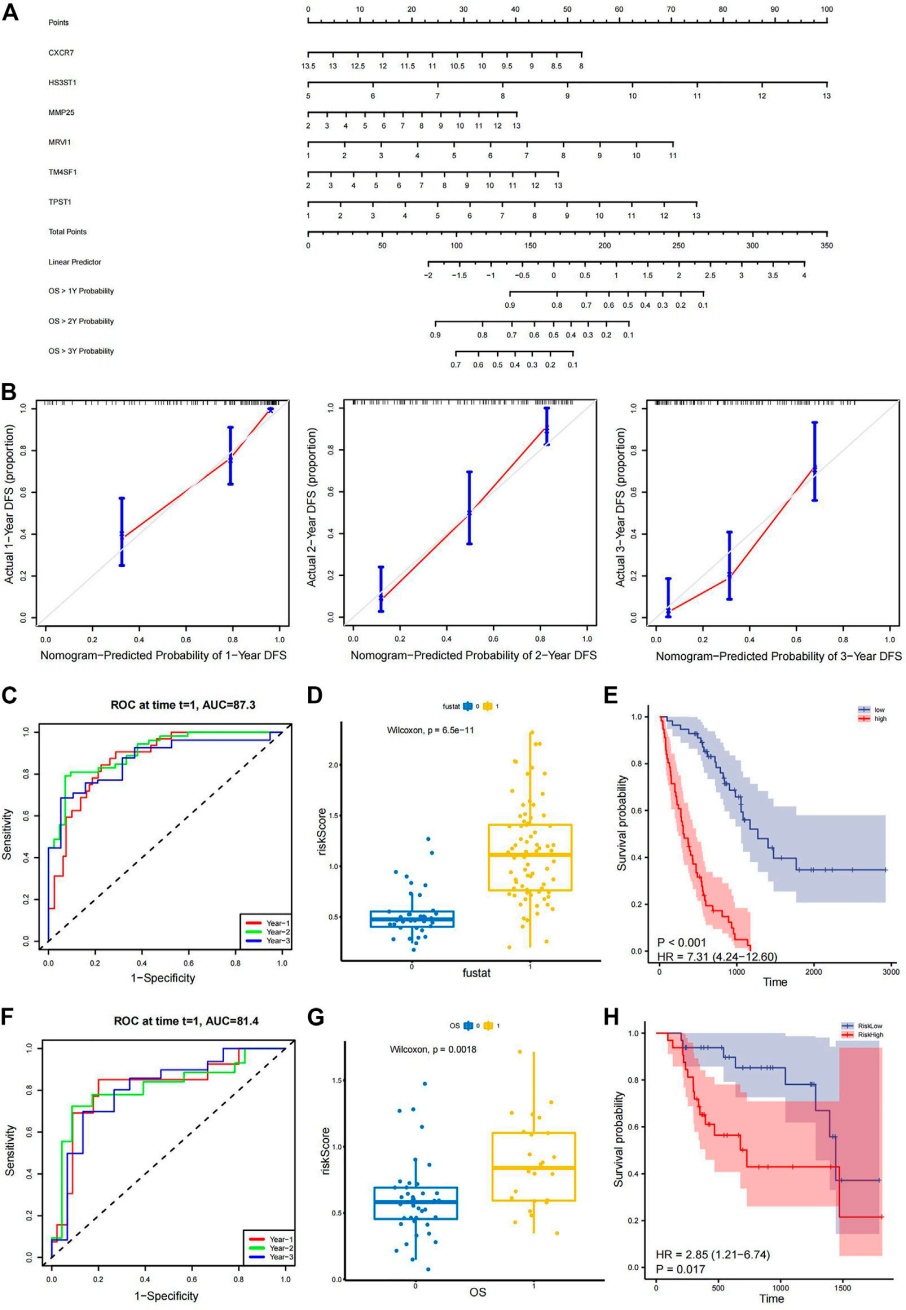
Evaluation and validation of prognostic models. **(A)** Nomogram of the model for 1-, 2-, and 3-year overall survival rates. **(B)** Calibration curves of the model based on 1-, 2-, and 3-year overall survival rates. **(C)** Time-dependent ROC curve based on the median of risk score in the training set. The 1-year AUC is .727, the 2-year AUC is .905, and the 3-year AUC is .868. **(D)** Box plots showing that the Wilcoxon *P*-test results (*P* = 6e-11) are less than .05 between the different groups based on the median of risk score in the training set. **(E)** Kaplan–Meier survival curve showing a clear difference between groups based on the median of risk score in the training set [HR = 7.31, 95% CI: (4.24, 12.60), *p* < .001]. **(F)** Time-dependent ROC curve based on the validation set. The 1-year AUC is .814, the 2-year AUC is .814, and the 3-year AUC is .808. **(G)** Box plots presenting a significant difference (*p* = .0018) in the validation set. **(H)** Kaplan–Meier survival curve showing a clear difference in the validation set [HR = 2.85, 95% CI: (1.21, 6.74), *p* = .017]. ROC, receiver operating characteristic; AUC, area under the curve; HR, hazard ratio; CI, confidence interval.

A nomogram was constructed using the training set, which was used to generate the prognostic model (Figure 7A). Calibration curves were also plotted for 1-, 2-, and 3-year overall survival rates (Figure 7B). To test the effect of our model, patients were divided into high-risk and low-risk groups according to the median value of the risk score in the training and validation sets (He et al., 2022; Lin et al., 2022). The risk curve (Supplementary Figure S2A) and the survival distribution figure (Supplementary Figure S2B) were plotted for the training set. The threshold value of the training set was .839, and there were 56 high-risk patients and 56 low-risk patients in the training set. The risk curve (Supplementary Figure S2C) and the survival distribution figure (Supplementary Figure S2D) were plotted for the validation set. The threshold value of the validation set was .615, and there were 32 high-risk patients and 32 low-risk patients in the validation set. The time-dependent ROC curves were plotted. In the training set, the 1-year AUC was .872, the 2-year AUC was .905, and the 3-year AUC was .868 (Figure 7C). In the validation set, the 1-year AUC was .814, the 2-year AUC was .814, and the 3-year AUC was .808 (Figure 7F), suggesting that the model had good predictive ability. Box plots and survival curves were also generated for the training and validation sets, respectively. The box plots showed that the Wilcoxon *p*-values were less than .05 for the training (*P* = 6e-11; Figure 7D) and validation sets (*p* = .0018; Figure 7G), indicating a significant difference between high- and low-risk patients in terms of prognosis. Survival analysis showed that the prognostic outcomes were poorer for high-risk patients in the training [HR = 7.31, 95% confidence interval (CI): (4.24, 12.60), *p* < .001; Figure 7E] and validation sets [HR = 2.85, 95% CI: (1.21, 6.74), *p* = .017; Figure 7H]. Two clinical factors (age and sex) were obtained for multivariate Cox analysis with the model (Table 1), revealing that both the training set risk score [HR = 13, 95% CI: (7.61, 22.9), *p* < .001] and validation set risk score [HR = 9.8, 95% CI: (2.79, 34.4), *p* < .001] had independent prognostic power.

**TABLE 1:** Multivariate Cox analysis of the training and validation sets.

Multivariate cox analysis		Training set	Validation set
Age	Hazard Ratio	1	1
	*p*-value	.456	.159
Sex	Hazard Ratio	1	1.1
	*p*-value	.991	.844
Risk score	Hazard Ratio	13	9.8
	*p*-value	<.001	<.001

## 4 Discussion

IPF is a disease with poor prognoses and a variable and unpredictable natural course. For prognostic outcomes of patients with IPF, prediction methods are mainly based on clinical symptoms and exposure, imaging, and histopathology (Lynch et al., 2018). However, these prediction methods have limited accuracy and a few are invasive; Thus, developing more accurate and safer methods for determining IPF prognosis is an urgent unmet need in clinical practice. With the development of bioinformatics, genomics and transcriptomics are becoming increasingly important to identify clinical predictive biomarkers (Kraaijvanger et al., 2020). Many studies have screened genes as novel biomarkers of common biological processes in IPF using bioinformatics. A study have screened novel prognostic markers based on cellular senescence characteristics in IPF (He and Li, 2022), whereas another study have screened new prognostic markers associated with ferroptosis characteristics in IPF (He et al., 2022). One study has shown that EMT plays an important role in IPF development, and in this study, an EMT-related prognostic model has been constructed using blood samples (Zheng et al., 2022), but the evaluation capacity of this model is limited, and the AUC values for both the training and validation sets are less than .80. Therefore, in the current study, the new perspective of EMT was used, and six novel prognostic biomarkers with higher accuracy were identified using BAL cell samples.

First, DEGs in BAL cells from normal and IPF samples were obtained, and then, their intersection with EMT-related genes from the MSigDB was determined to obtain differentially expressed EMT-related genes (*TIMP3*, *SPP1*, *ITGB3*, and *HTRA1*). The results indicated a link between EMT and IPF development. The samples and the four obtained EMT-related genes were further analyzed in depth. TIMP3 is highly expressed in lung fibroblasts, is induced by transforming growth factor-β1, and may be an important mediator of lung fibrosis (García-Alvarez et al., 2006). Regarding SPP1, macrophages expressing high levels of this marker have important effects on pulmonary fibrosis (Morse et al., 2019). Blocking SPP1 expression in mice inhibits the development of pulmonary fibrosis (Kumar et al., 2022). ITGB3 plays an important role in vesicle uptake and is closely associated with tumor metastasis (Fuentes et al., 2020). HTRA1 is closely related to growth factor β, NOTCH, and other signaling pathways and plays an important role in cell migration and proliferation (Oka et al., 2022). Using the correlation and immune cell infiltration analyses, we found that *HTRA1* and *SPP1* may be positively correlated with the EMT process and that EMT-related genes may be closely associated with immune dysregulation, especially that pertaining to activated mast cells in IPF. A previous animal experiment has also demonstrated the correlation between IPF and activated mast cells. Accordingly, mast cell deficiency reduces pulmonary fibrosis (Veerappan et al., 2013). Therefore, it is valuable to screen EMT-related genes in IPF for clinical and basic research.

We also performed a consensus clustering analysis of IPF samples and classified patients with IPF into clusters 1 and 2 based on the differential expression of EMT-related genes; then, PCA was used to verify the accuracy of the consensus clustering results. Biological functions and pathways that differed between clusters 1 and 2 were investigated using GSVA. The differential functions and pathways identified were mainly related to metabolism and immunity, suggesting that EMT might aggravate IPF development through metabolic abnormalities. WGCNA was further performed on the consensus clustering results, and 239 genes most associated with EMT were obtained, allowing the identification of EMT-related candidate genes to construct prognostic models. LASSO and Cox regression analyses were then performed to obtain six genes that were closely related to prognosis, allowing the construction of a prognostic model and a risk score formula. The model was presented and evaluated based on the nomogram plot and calibration curves. Survival, ROC, and multivariate Cox analyses on the training and validation sets were performed. The model better differentiated patients according to their prognostic outcomes. In addition, the AUC values for the training and validation sets for 1-, 2-, and 3-year overall survival rates were greater than .80, demonstrating that this model exhibits considerably better performance than the previous model (Zheng et al., 2022) and suggesting that this prognostic model has better predictive power.

A total of six genes were screened in the prognostic model (*CXCR7*, *HS3ST1*, *MMP25*, *MRVI1*, *TM4SF1*, and *TPST1*). *CXCR7* (updated as *ACKR3*) encodes atypical chemokine receptor 3, which binds to a variety of endogenous and exogenous ligands, such as stromal cell-derived factor 1 and macrophage migration inhibitory factor (Wang et al., 2018). CXCR7 activates signaling pathways, such as mitogen-activated protein kinase (Rajagopal et al., 2010; Heinrich et al., 2012), and SDF-1/CXCR4 activation affects IPF development (Amano et al., 2019). *HS3ST1* encodes a member of the heparan sulfate biosynthetic enzyme family. HS3TA is closely related to inflammation and metabolism and is significantly associated with the fibrosis developmental process (Ferreras et al., 2019). *MMP25* encodes a member of the matrix metalloproteinase (MMP) family, and MMP25 deficiency may lead to immune abnormalities in mice (Soria-Valles et al., 2016). Further, MMP25 may be strongly associated with cancer development and the progression of other diseases by affecting immune functions (Sohail et al., 2008). *MRVI1* encodes a protein whose expression is closely related to nasopharyngeal and colorectal cancer (Zhu et al., 2019; Ma et al., 2020). MRVI1 acts as a nitric oxide/protein kinase cGMP-dependent 1-dependent regulator that regulates intracellular Ca^2+^ to affect physiological functions of the organism (Schlossmann et al., 2000). *MRVI1* might also be associated with IPF progression. *TM4SF1* encodes a transmembrane four superfamily protein, which affects fibroblast motility, proliferation, and apoptosis through pathways, such as protein kinase B/extracellular signal-regulated kinases (Xu et al., 2020). *TM4SF1* is associated with diseases, such as non-small cell lung cancer and gastric cancer (Peng et al., 2018; Fu et al., 2020). Its role in cell motility (Zukauskas et al., 2011) may be related to fibroblast migration during IPF development. *TPST1* encodes tyrosylprotein sulfotransferase 1, which affects inflammatory and immune responses by altering protein activity (Šmak et al., 2021); thus, *TPST1* might be associated with IPF progression. Studies on EMT-related prognostic genes in the context of IPF are insufficient. Thus, future studies need to identify and experimentally validate EMT-related genes as IPF prognostic genes.

The prediction accuracy of the constructed prognostic model was relatively high, with a 2-year AUC in the training set of .905 and a 2-year AUC in the validation set of .814. Owing to a lack of EMT-related prognostic models, this study provides reference values for the clinical translation of EMT targets for IPF. There were some limitations to the study. First, only a limited number of samples were included in the study. Because the study was conducted based on comprehensive bioinformatics, genes of interest were not experimentally validated. In the future, the possible mechanisms of the identified EMT genes will be explored through clinical and experimental approaches.

## 5 Conclusion

Here, EMT-related genes in IPF were determined. Through bioinformatics analyses, six genes were identified that were closely related to IPF prognosis and were used to construct a prognostic model. This model better assessed the prognosis of IPF, which might promote the translation of basic research on EMT to clinical strategies for disease treatment. We hypothesize that this model may improve IPF clinical diagnosis and treatment in the future.

## Data Availability

The original contributions presented in the study are included in the article/Supplementary Material, further inquiries can be directed to the corresponding author.

## Abbreviations

LASSO, least absolute shrinkage and selection operator; ROC, receiver operating characteristic; GEO, Gene Expression Omnibus; IPF, idiopathic pulmonary fibrosis; EMT, epithelial–mesenchymal transition; BAL, bronchoalveolar lavage; BALF, bronchoalveolar lavage fluid; WGCNA, weighted gene co-expression network analysis; KEGG, Kyoto Encyclopedia of Genes and Genomes; DEGs, differentially expressed genes; GO, Gene Ontology; CDF, cumulative distribution function; GSVA, gene set variation analysis; TOM, topological overlap matrix; HR, hazard ratio; AUC, area under the curve; PCA, principal component analysis.
