# Supplementary material for: A prognostic model based on clusters of molecules related to epithelial–mesenchymal transition for idiopathic pulmonary fibrosis
Source: Front Genet. 2023 Jan 6;13:1109903. doi: 10.3389/fgene.2022.1109903 (PMC9853015; doi:10.3389/fgene.2022.1109903)
Supplement: Supplementary file 1 [file DataSheet1.pdf]

Supplementary Material

A prognostic model based on clusters of molecules related to epithelial–mesenchymal transition for idiopathic pulmonary fibrosis

Jiarui Zhao[1], Can Wang[1], Rui Fan[1], Xiangyang Liu[1], Wei Zhang[2*]

[1]Shandong University of Traditional Chinese Medicine, Jinan, Shandong, China

[2]College of First Clinical Medicine, Shandong University of Traditional Chinese Medicine, Jinan, China

*** Correspondence:**

Wei Zhang

huxizhijia@126.com

1 Supplementary Table

1.1 Supplementary Table 1. The Clinical information of the training set

ID	sex (0=female, 1=male)	age	survival status (0 = censored, 1 = death)	time to death (year)
GSM1820739	1	61	0	8.016438356

GSM1820740	1	70	1	0.835616438
GSM1820741	1	74	1	1.526027397
GSM1820742	1	55	1	0.41369863
GSM1820743	1	47	1	3.846575342
GSM1820744	1	47	1	1.967123288
GSM1820745	1	65	1	0.457534247
GSM1820746	1	50	1	2.887671233
GSM1820747	1	53	1	1.57260274
GSM1820748	0	61	1	0.542465753
GSM1820749	0	51	1	2.961643836
GSM1820750	1	61	1	2.690410959
GSM1820751	0	66	1	3.484931507
GSM1820752	1	71	0	5.895890411
GSM1820753	1	70	1	2.221917808

GSM1820754	1	62	0	6.843835616
GSM1820755	1	70	1	0.265753425
GSM1820756	1	68	1	0.224657534
GSM1820757	1	62	1	2.257534247
GSM1820758	0	61	1	1.698630137
GSM1820759	1	65	0	5.383561644
GSM1820760	1	76	0	6.128767123
GSM1820761	1	68	1	1.021917808
GSM1820762	1	56	1	2.556164384
GSM1820763	0	70	0	5.460273973
GSM1820764	1	77	1	3.221917808
GSM1820765	1	54	1	0.605479452
GSM1820766	1	69	1	2.671232877
GSM1820767	1	49	1	4.04109589

GSM1820768	1	79	1	4.846575342
GSM1820769	1	78	1	0.75890411
GSM1820770	1	58	1	3.123287671
GSM1820771	1	74	0	4.967123288
GSM1820772	1	76	1	1.183561644
GSM1820773	1	75	1	2.479452055
GSM1820774	1	74	1	2.446575342
GSM1820775	1	76	0	4.328767123
GSM1820776	1	72	0	4.002739726
GSM1820777	1	76	1	2.890410959
GSM1820778	0	72	1	0.123287671
GSM1820779	1	56	1	1.498630137
GSM1820780	1	63	0	2.660273973
GSM1820781	1	50	1	1.64109589

GSM1820782	0	67	1	1.465753425
GSM1820783	1	77	1	0.161643836
GSM1820784	1	65	1	0.575342466
GSM1820785	1	78	0	1.912328767
GSM1820786	1	63	1	0.619178082
GSM1820787	1	53	0	1.835616438
GSM1820788	0	73	1	0.268493151
GSM1820789	1	74	0	1.742465753
GSM1820790	1	75	0	1.679452055
GSM1820791	1	72	0	1.62739726
GSM1820792	1	70	1	0.838356164
GSM1820793	1	77	1	0.219178082
GSM1820794	1	54	1	1.547945205
GSM1820795	0	68	1	0.410958904

GSM1820796	1	74	0	1.432876712
GSM1820797	1	60	0	2.298630137
GSM1820798	1	61	1	0.180821918
GSM1820799	1	76	1	1.339726027
GSM1820800	1	68	1	0.057534247
GSM1820801	1	67	1	0.243835616
GSM1820802	1	77	0	1.509589041
GSM1820803	1	66	1	0.350684932
GSM1820804	1	59	0	1.282191781
GSM1820805	0	54	1	2.301369863
GSM1820806	1	63	1	0.539726027
GSM1820807	0	67	1	1.293150685
GSM1820808	0	71	0	2.389041096
GSM1820809	0	84	1	2.156164384

GSM1820810	1	69	0	2.887671233
GSM1820811	1	71	0	2.882191781
GSM1820812	1	48	1	1.035616438
GSM1820813	1	67	1	1.884931507
GSM1820814	1	71	1	1.350684932
GSM1820815	1	78	1	2.624657534
GSM1820816	1	69	0	2.364383562
GSM1820817	1	65	1	1.605479452
GSM1820818	0	44	0	3.02739726
GSM1820819	1	68	1	3.208219178
GSM1820820	1	53	1	0.301369863
GSM1820821	1	51	1	1.293150685
GSM1820822	1	60	1	0.380821918
GSM1820823	0	49	0	2.304109589

GSM1820824	1	83	1	0.805479452
GSM1820825	1	54	1	1.063013699
GSM1820826	1	82	1	0.871232877
GSM1820827	1	82	0	3.024657534
GSM1820828	0	68	0	3.005479452
GSM1820829	1	81	1	0.115068493
GSM1820830	1	75	1	0.756164384
GSM1820831	1	74	1	0.216438356
GSM1820832	1	62	0	2.81369863
GSM1820833	1	76	1	1.526027397
GSM1820834	1	66	0	1.808219178
GSM1820835	1	86	1	0.394520548
GSM1820836	1	70	1	0.742465753
GSM1820837	1	62	0	1.695890411

GSM1820838	0	81	0	2.353424658
GSM1820839	0	70	0	1.597260274
GSM1820840	0	78	1	2.002739726
GSM1820841	1	64	0	1.504109589
GSM1820842	1	79	0	1.334246575
GSM1820843	1	66	1	1.479452055
GSM1820844	1	62	1	0.77260274
GSM1820845	1	61	0	1.257534247
GSM1820846	1	73	1	1.167123288
GSM1820847	1	81	1	1.098630137
GSM1820848	1	68	0	1.049315068
GSM1820849	1	72	1	1.010958904
GSM1820850	1	84	1	0.668493151

1.2 Supplementary Table 2. The Clinical information of the validation set

ID	sex (0=female, 1=male)	age	survival status (0 = censored, 1 = death)	time to death (year)
GSM1820851	0	74	1	3.953424658
GSM1820852	1	64	0	1.608219178
GSM1820853	1	64	1	0.58630137
GSM1820854	1	58	0	0.991780822
GSM1820855	1	67	1	1.282191781
GSM1820856	1	63	1	1.852054795
GSM1820857	1	60	0	3.895890411
GSM1820858	1	64	1	4.032876712
GSM1820859	1	71	0	1.18630137
GSM1820860	0	76	0	3.482191781
GSM1820861	1	66	1	3.509589041

GSM1820862	1	65	0	1.915068493
GSM1820863	1	68	1	0.961643836
GSM1820864	1	77	0	0.898630137
GSM1820865	1	53	0	1.449315068
GSM1820866	1	64	0	2.317808219
GSM1820867	1	75	0	1.915068493
GSM1820868	0	62	0	1.147945205
GSM1820869	1	52	0	3.432876712
GSM1820870	1	72	0	1.909589041
GSM1820871	0	72	0	3.397260274
GSM1820872	1	71	1	3.819178082
GSM1820873	1	51	0	3.942465753
GSM1820874	1	60	1	1.745205479
GSM1820875	1	66	0	2.515068493

GSM1820876	1	74	0	4.978082192
GSM1820877	1	64	1	0.819178082
GSM1820878	1	69	0	1.032876712
GSM1820879	0	80	0	1.495890411
GSM1820880	1	74	0	2.076712329
GSM1820881	1	73	1	1.482191781
GSM1820882	1	75	1	0.254794521
GSM1820883	0	69	0	2.471232877
GSM1820884	1	78	1	1.082191781
GSM1820885	1	87	0	1.821917808
GSM1820886	0	61	0	3.41369863
GSM1820887	0	79	1	0.602739726
GSM1820888	1	71	1	0.58630137
GSM1820889	1	64	0	0.953424658

GSM1820890	1	52	1	0.547945205
GSM1820891	1	60	1	0.693150685
GSM1820892	1	62	0	2.260273973
GSM1820893	0	77	0	1.065753425
GSM1820894	1	73	0	1.01369863
GSM1820895	0	65	0	1.531506849
GSM1820896	1	76	0	1.57260274
GSM1820897	1	51	0	3.849315068
GSM1820898	1	78	1	0.81369863
GSM1820899	0	53	0	0.665753425
GSM1820900	1	80	0	0.652054795
GSM1820901	1	69	0	0.649315068
GSM1820902	1	77	1	0.838356164
GSM1820903	1	69	1	2.843835616

GSM1820904	0	69	0	2.452054795
GSM1820905	1	63	0	4.912328767
GSM1820906	1	65	0	1.052054795
GSM1820907	1	68	0	1.150684932
GSM1820908	1	53	0	4.084931507
GSM1820909	1	82	1	0.646575342
GSM1820910	1	79	0	3.008219178
GSM1820911	0	67	1	0.931506849
GSM1820912	1	79	1	1.994520548
GSM1820913	1	80	1	0.364383562
GSM1820914	1	68	0	2.57260274

1.3 Supplementary Table 3. The differentially expressed genes

Gene.symbol	LogFC	AveExpr	t	P.Value	adj.P.Val	B	Plog	Type
CYTL1	2.379062232	5.656305505	8.628687372	1.60E-14	3.23E-10	22.21765592	9.49066207	Diff

SPP1	3.856307039	10.73748932	8.067517563	3.63E-13	2.92E-09	19.28308569	8.535145073	Diff
FAM125B	-2.01535831	10.07718164	-8.026336292	4.55E-13	2.92E-09	19.07001126	8.535145073	Diff
PPBP	3.766861875	11.28133473	7.982838301	5.78E-13	2.92E-09	18.84532425	8.535145073	Diff
TCF7L1	-2.032309964	4.024233352	-7.685401743	2.92E-12	1.07E-08	17.31984298	7.970303475	Diff
GFRA2	1.757832762	9.643176508	7.669822483	3.18E-12	1.07E-08	17.24049337	7.970303475	Diff
CAMP	-1.834538032	10.91891479	-7.194833959	4.04E-11	1.04E-07	14.85068692	6.98224743	Diff
TCEA3	-1.917583098	8.607141451	-7.190644798	4.13E-11	1.04E-07	14.8298806	6.98224743	Diff
ITGB3	2.776961403	5.67814492	7.072071412	7.70E-11	1.41E-07	14.24307559	6.849557235	Diff
CST6	1.888113842	6.871769818	6.854799168	2.39E-10	3.58E-07	13.17886172	6.445656808	Diff
MATK	1.745332819	7.476377399	6.847208413	2.49E-10	3.58E-07	13.14195054	6.445656808	Diff
FABP3	2.102836398	9.854238563	6.749001022	4.12E-10	5.55E-07	12.66610057	6.255748908	Diff
ENPP3	-2.05389966	4.8718809	-6.584005659	9.58E-10	1.07E-06	11.87395548	5.968801657	Diff
PLA2G7	2.106076628	9.855915136	6.503586664	1.44E-09	1.45E-06	11.49131538	5.837646034	Diff
ZNF702P	-1.662616021	3.656883383	-6.413943295	2.26E-09	2.17E-06	11.06754918	5.662857675	Diff

TMEM56	-1.58702523	3.749365463	-6.38895936	2.56E-09	2.35E-06	10.949974	5.628676446	Diff
RNASE2	1.595823722	7.643970795	6.356004772	3.02E-09	2.54E-06	10.79524749	5.594888952	Diff
EMP1	1.73448369	12.36990663	6.331938034	3.41E-09	2.65E-06	10.68251078	5.577494191	Diff
MMP7	3.094390723	7.461438112	6.290465961	4.19E-09	3.09E-06	10.48876193	5.509883416	Diff
CXCR7	-1.655972404	11.02267261	-6.285762646	4.29E-09	3.09E-06	10.4668309	5.509883416	Diff
NALCN	-2.560818677	5.825051918	-6.266246222	4.72E-09	3.18E-06	10.37591975	5.49777675	Diff
LOC645206	-1.554629097	3.525140909	-6.169928997	7.60E-09	4.30E-06	9.929447914	5.366880599	Diff
SPRY2	1.585723351	6.981320866	6.155223718	8.17E-09	4.43E-06	9.861607549	5.354060081	Diff
C14orf34	2.130256689	4.939422818	6.072301034	1.23E-08	6.35E-06	9.480699824	5.197271343	Diff
CYP3A7	-1.730939185	5.497746839	-6.063489573	1.28E-08	6.46E-06	9.440389816	5.189596688	Diff
ITIH5	-2.261720376	5.951593169	-6.053517223	1.34E-08	6.62E-06	9.394807833	5.179207823	Diff
SFTPB	3.00199104	8.646018845	6.009377168	1.67E-08	8.01E-06	9.193548325	5.096441718	Diff
AOC3	-1.880362177	11.69843577	-5.982917817	1.89E-08	8.69E-06	9.073297221	5.060930453	Diff
TIMP3	2.238785546	5.276087624	5.925748385	2.50E-08	1.10E-05	8.814492227	4.960301205	Diff

CYP1B1	2.489640251	7.968783826	5.885746719	3.03E-08	1.30E-05	8.634240868	4.886088806	Diff
ARAP3	1.624521865	8.018251334	5.799997501	4.56E-08	1.77E-05	8.250204223	4.751919635	Diff
EFCAB1	-1.603688356	3.69990404	-5.788414958	4.82E-08	1.80E-05	8.198580243	4.744365819	Diff
HTRA1	2.24098449	7.707990798	5.715006133	6.82E-08	2.42E-05	7.872796602	4.61670383	Diff
C8B	-2.31630506	11.3093596	-5.694362706	7.52E-08	2.53E-05	7.78162326	4.59666936	Diff
RNASE1	1.678980258	12.97792378	5.642279686	9.61E-08	3.13E-05	7.552465851	4.504540005	Diff
PGA3	1.851338328	4.14190845	5.638166389	9.80E-08	3.14E-05	7.534421506	4.503111551	Diff
STAB1	1.966869922	7.403141806	5.515551033	1.73E-07	5.00E-05	7.000185141	4.300736306	Diff
F13A1	1.651879634	8.373060922	5.463049799	2.21E-07	6.29E-05	6.773634233	4.201606406	Diff
GBP7	-1.72055309	4.467536921	-5.438132556	2.48E-07	6.86E-05	6.666581573	4.163903406	Diff
VSNL1	2.263340689	4.81940438	5.381225851	3.22E-07	8.44E-05	6.423236922	4.07390728	Diff
FFAR3	2.236114239	5.468092103	5.352396944	3.67E-07	9.50E-05	6.300572263	4.022448136	Diff
TPSAB1	2.048685436	7.665935715	5.325285744	4.15E-07	0.000106052	6.185596386	3.974482086	Diff
DLX4	-1.552130689	5.918468883	-5.312367076	4.40E-07	0.000109672	6.130939954	3.959903991	Diff

SPINK1	2.068831852	7.492122701	5.238002123	6.15E-07	0.000139604	5.817967495	3.855103121	Diff
MERTK	2.137822409	6.244214434	5.219524212	6.69E-07	0.00014834	5.740641634	3.828740285	Diff
TUBB3	2.463030969	5.554210786	5.138633641	9.59E-07	0.00019175	5.404225211	3.717263858	Diff
PID1	1.667189578	5.637877591	5.093193386	1.17E-06	0.00022135	5.21675507	3.654919965	Diff
OLIG1	2.237908833	6.08214498	5.067748613	1.31E-06	0.000240884	5.11225982	3.618191738	Diff
PRRT4	-1.55287501	7.084476506	-5.061408844	1.35E-06	0.00024547	5.086278024	3.610002037	Diff
CH25H	1.575410606	8.328110632	5.022974215	1.60E-06	0.000276038	4.929228307	3.559030849	Diff
CYP3A5	-2.163955841	4.755146794	-5.009838756	1.69E-06	0.000286623	4.875738305	3.542688264	Diff
LOC400043	-1.630376424	7.022148464	-4.989876058	1.85E-06	0.000303054	4.794626484	3.518479617	Diff
SDS	1.879721761	7.963068667	4.981277206	1.92E-06	0.000304721	4.759755003	3.516097892	Diff
RGL1	1.550589199	7.559074839	4.900530506	2.72E-06	0.000392286	4.434285518	3.406396682	Diff
CPA3	1.649795797	7.268772607	4.88617701	2.89E-06	0.000411592	4.376809319	3.385533599	Diff
MURC	-1.628896	4.618241047	-4.886090341	2.89E-06	0.000411592	4.376462616	3.385533599	Diff
SOD3	1.973454251	4.665393822	4.84755715	3.42E-06	0.000462844	4.222736446	3.334565788	Diff

TPSD1	1.936441999	7.254015366	4.817595424	3.88E-06	0.000515713	4.103785045	3.287591628	Diff
SFTPC	1.939077383	10.51554479	4.81079394	4.00E-06	0.000523998	4.076853381	3.280670488	Diff
LOC100128252	-1.578528872	4.583905179	-4.78386928	4.48E-06	0.000564681	3.970499498	3.24819687	Diff
SLC28A3	1.605449214	6.190798146	4.77676558	4.62E-06	0.000574047	3.942508653	3.241052856	Diff
CCL2	2.199945765	11.90312816	4.773567445	4.68E-06	0.000576448	3.929916429	3.239239821	Diff
SEPP1	1.529309801	8.544595582	4.735917226	5.49E-06	0.000648333	3.782116597	3.188201965	Diff
LRRC2	2.41023501	7.171372645	4.710401546	6.11E-06	0.000713574	3.682418426	3.146560858	Diff
RAB3IL1	1.533627164	4.844205659	4.704901071	6.26E-06	0.000721924	3.660975861	3.141508684	Diff
MMP10	2.15791477	4.539991178	4.666796388	7.34E-06	0.000792613	3.512917462	3.100938646	Diff
ANGPTL4	1.768238304	4.845903659	4.614893366	9.11E-06	0.000910878	3.31261919	3.040539978	Diff
B3GNT8	1.593424928	8.023898235	4.534631945	1.27E-05	0.001118018	3.00604256	2.951551185	Diff
KCNAB1	-1.707621704	6.821786692	-4.534466583	1.27E-05	0.001118018	3.00541492	2.951551185	Diff
RSPH1	-1.525034313	5.300816379	-4.467637851	1.67E-05	0.001335951	2.753122952	2.87420939	Diff
COL22A1	1.676616067	4.502948094	4.431280537	1.93E-05	0.001435257	2.617014299	2.843070443	Diff

VSTM1	1.917150621	7.753890122	4.422789332	2.00E-05	0.001469136	2.585343674	2.832938134	Diff
CCR3	1.661583017	4.997643457	4.418056217	2.04E-05	0.001477945	2.567709392	2.830341855	Diff
BICC1	2.109563647	4.382100528	4.363078927	2.54E-05	0.001758835	2.363899093	2.754774944	Diff
OR13H1	1.517023243	3.816244219	4.34757335	2.71E-05	0.001839751	2.306758535	2.735240865	Diff
FNDC5	2.003284599	5.418493581	4.340982281	2.78E-05	0.001882425	2.282515098	2.725282214	Diff
CCL7	1.932008894	7.286022705	4.336940484	2.82E-05	0.001887659	2.26766197	2.724076549	Diff
GABRE	-1.722055061	6.465204571	-4.309665792	3.15E-05	0.002043	2.167699965	2.689731628	Diff
MGC24103	1.581077659	4.599248099	4.306654357	3.18E-05	0.002051551	2.156691844	2.687917598	Diff
LEF1	-1.85637229	5.372125495	-4.170053993	5.44E-05	0.002946191	1.663449652	2.530739098	Diff
CD86	1.637617807	10.11183645	4.128339803	6.40E-05	0.003277439	1.51523063	2.484465422	Diff
PTGER3	-1.999655446	5.674523512	-4.099639958	7.14E-05	0.003483005	1.413916145	2.458045887	Diff
GPR179	2.106137851	5.812858902	4.080184546	7.69E-05	0.003646715	1.345544417	2.438098123	Diff
NT5DC2	1.601696863	5.678319341	4.076912983	7.79E-05	0.003651107	1.334071803	2.43757542	Diff
TM4SF1	1.762116153	6.246382024	4.06326721	8.21E-05	0.003774827	1.286295712	2.423102924	Diff

TMEM200A	-1.544344727	4.541998817	-4.037945135	9.04E-05	0.003992837	1.197966658	2.398718369	Diff
IL1R2	2.000879553	6.254742864	3.96478792	0.000119101	0.004761686	0.945187168	2.322239262	Diff
DMD	-1.525466814	4.659086169	-3.908573551	0.000146886	0.005574927	0.753405266	2.253760778	Diff
RPA4	1.625584253	7.729595477	3.877431528	0.000164832	0.005996309	0.648087952	2.222115962	Diff
DNAI2	-1.638820302	5.011994674	-3.813628822	0.000208319	0.007068831	0.434401112	2.150652403	Diff
PROM2	1.904577887	6.056509782	3.795580285	0.000222474	0.00737562	0.374464818	2.132201482	Diff
FAM183A	-1.561556157	4.934698584	-3.778522984	0.000236688	0.00760943	0.318028807	2.118647878	Diff
CXCL14	1.579118233	4.297626916	3.759903979	0.000253184	0.007987175	0.256657786	2.097606792	Diff
AQP4	1.980849371	4.917231727	3.749133936	0.000263219	0.0082266	0.221268993	2.084779603	Diff
S100A12	2.119290789	7.241657268	3.743050707	0.000269052	0.008331524	0.201316374	2.07927556	Diff
NRAP	1.671993105	4.444435047	3.739861849	0.000272158	0.008389119	0.190867505	2.076283636	Diff
CD40LG	-1.873080533	6.813476324	-3.722279198	0.000289906	0.008841697	0.133383319	2.053464374	Diff
DACH1	1.676198748	4.951112706	3.711779386	0.000301022	0.009071086	0.099159515	2.042340702	Diff
MRVI1	1.542055542	5.398381108	3.665552174	0.000354938	0.010207157	-0.050588102	1.991095211	Diff

TPST1	1.913459363	5.307353682	3.619950215	0.000416982	0.011423145	-0.196818967	1.942214325	Diff
IGF1	-1.541707205	7.261456858	-3.562679347	0.000509453	0.012970803	-0.378352592	1.887033142	Diff
DEFA3	2.096240137	6.982338525	3.548442647	0.000535272	0.013298298	-0.42311125	1.876203951	Diff
GPR182	1.869562833	5.39909	3.523230502	0.000584046	0.014037964	-0.502014158	1.85269586	Diff
EHD2	1.594391304	5.284562094	3.522395312	0.000585731	0.014045029	-0.504620012	1.852477365	Diff
HS3ST2	1.835975538	5.832087198	3.522037638	0.000586454	0.014045686	-0.505735826	1.852457038	Diff
AANAT	1.710072135	3.90009583	3.455761242	0.00073595	0.01636435	-0.710878049	1.786101234	Diff
WNT2B	1.87467002	4.493496423	3.179253963	0.001835104	0.02952577	-1.531418168	1.52979877	Diff
LOC729040	1.764732381	4.044692615	3.129669494	0.002149249	0.032313674	-1.672414127	1.490613657	Diff
KIAA0125	1.652980521	4.586970228	3.103458075	0.002334793	0.034028076	-1.746179169	1.468162607	Diff
HBD	1.867476983	11.72075912	2.983028127	0.003393543	0.043227527	-2.078202178	1.364239606	Diff

1.4 Supplementary Table 4. The EMT-related genes from the Molecular Signatures Database

COL3A1

COL5A2

COL5A1

FBN1

COL1A1

FN1

COL6A3

SERPINE1

COL1A2

COL4A1

COL4A2

VCAN

IGFBP3

TGFBI

SPARC

LUM

LAMC1

LOX

LAMC2

CCN2

TAGLN

COL7A1

LOXL2

COL6A2

ITGAV

THBS2

COL16A1

NNMT

TPM1

CDH2

MMP2

COL11A1

THBS1

FAP

BGN

SERPINH1

FSTL1

POSTN

THY1

SPP1

TNC

TFPI2

NID2

ITGB5

MMP3

VIM

LOXL1

FBLN5

COL12A1

ELN

CDH11

COMP

SPOCK1

BMP1

IL32

LAMA3

TIMP1

QSOX1

TIMP3

VCAM1

CCN1

EDIL3

CALD1

MAGEE1

FBLN1

SGCB

ECM1

LAMA2

FSTL3

TPM2

INHBA

DAB2

EMP3

BASP1

ITGA5

MGP

VEGFA

CXCL1

WNT5A

SDC1

PLOD2

PCOLCE

GREM1

ITGB1

COL5A3

RHOB

HTRA1

FGF2

SNTB1

GADD45A

MEST

LRRC15

TNFRSF11B

CD59

ACTA2

EFEMP2

MATN2

PCOLCE2

SERPINE2

GPC1

ABI3BP

FUCA1

SLIT3

LAMA1

PMEPA1

COL8A2

FBN2

IGFBP2

PFN2

SDC4

CD44

GADD45B

CXCL8

GLIPR1

ANPEP

P3H1

VEGFC

MMP14

SGCD

PLOD1

MATN3

MYL9

SLC6A8

CALU

PRRX1

TNFRSF12A

FMOD

ID2

GEM

PLAUR

MYLK

TGFB1

SFRP1

PLOD3

IL6

APLP1

FBLN2

MSX1

PTX3

FZD8

JUN

FERMT2

DKK1

SNAI2

DST

TPM4

DCN

GJA1

PMP22

IGFBP4

COPA

LRP1

ITGA2

FLNA

MFAP5

PTHLH

TGFBR3

SFRP4

LGALS1

RGS4

CDH6

SAT1

NT5E

DPYSL3

PPIB

TGM2

SGCG

ITGB3

PDLIM4

CTHRC1

ECM2

CRLF1

AREG

IL15

MCM7

GAS1

PRSS2

CADM1

OXTR

SCG2

CXCL6

MMP1

TNFAIP3

CAPG

CAP2

MXRA5

FOXC2

NTM

ENO2

FAS

BDNF

ADAM12

PVR

CXCL12

PDGFRB

SLIT2

NOTCH2

COLGALT1

GPX7

WIPF1

1.5 Supplementary Table 5. The CIBERSORT scores

ID	B cells naive	B cells memory	Plasma cells	T cells CD8	T cells CD4 naive	T cells CD4 memory resting	T cells CD4 memory activated	T cells follicular helper	T cells regulatory (Tregs)	T cells gamma delta	NK cells resting	NK cells activated	Monocytes	Macrophages M0	Macrophages M1	Macrophages M2	Dendritic cells resting	Dendritic cells activated	Mast cells resting	Mast cells activated	Eosinophils	Neutrophils
GSM1820719_Control	0.015813735	0	0	0.051652717	0	0.113466752	0.023425376	0.007904995	0	0	0.006530205	0	0.03257115	0.345789199	0.007806855	0.318919692	0.009295864	0	0	0	0	0.066823461
GSM1820720_Control	0.019844936	0	0	0.029632139	0	0.116001865	0.007663596	0	0	0	0.004610852	0.00461852	0.043459248	0.469370586	0.006723045	0.234391338	0.012843556	0	0	0.005392332	0.005234461	0.040213527

GSM1820721_Control	0.02166358	0	0	0.034505091	0	0.072836961	0.033766209	0.013056071	0	0	0.010896562	0.005747414	0.056694289	0.413626822	0.025796252	0.225614701	0.015905243	0	0.009096958	0	0.004939931	0.060299915
GSM1820722_Control	0.01974565	0	0	0.035499757	0	0.114046425	0.011863121	0	0	0	0	0.039921757	0.032760376	0.243981737	0.030129484	0.313343987	0	0	0	0.045065824	0.021515618	0.09203038
GSM1820723_Control	0.027997151	0	0	0.057057373	0	0.063676156	0.020622313	0.012484253	0	0	0	0.034483179	0.1028077	0.315872208	0.01926695	0.260731588	0.015002992	0	0.002546474	0.005214156	0.008648462	0.053589046
GSM1820724_Control	0.016087175	0	0	0.023748989	0	0.108697124	0.027586758	0	0	0	0.028335024	0	0.121192304	0.472618424	0.00930402	0.127565269	0.012383641	0	0.00542094	0	0	0.047060333
GSM1820	0.01900	0	0	0.0361	0	0.106559143	0.034958714	0.004216069	0	0	0.0211	0	0.0918	0.34103	0.00884	0.22300	0.014765478	0	0.0169	0	0	0.0814

725_Control	9043			73983							8587		53573	5924	8462	4335			08954			80452
GSM1820726_Control	0.029226195	0	0	0.057882207	0	0.07151719	0.044779932	0.024427596	0	0	0	0.023680555	0.001818877	0.334489768	0.014908972	0.276216268	0.015957712	0	0.008124385	0	0.018759433	0.078210908
GSM1820727_Control	0.022774608	0	0	0.035195932	0	0.123101978	0.026591724	0.012850612	0	0	0.010517237	0.005746105	0.024539588	0.371223844	0.012940813	0.241063974	0.014402863	0	0	0.016866683	0.009519604	0.072664433
GSM1820728_Control	0.023076939	0	0	0.084089227	0	0.088915502	0.048047081	0.007598214	0	0	0	0.016850424	0.033587743	0.323421192	0.018835963	0.274938898	0.014044857	0	0	0.014885703	0.013641336	0.038066922
GSM1820729_Co	0.024158228	0	0	0.044417265	0.012814536	0.047206515	0.036646282	0.01718676	0	0	0.000959651	0.009405181	0.116007976	0.265031126	0.010173816	0.296820971	0.014768874	0	0	0.078933383	0.007785484	0.017683953

ntrol																						
GSM1820730_Control	0.016806047	0	0	0.090284697	0	0.059618493	0.129661409	0	0	0	0	0.022861602	0.020139729	0.249111749	0.0564465	0.287547874	0.001073861	0	0.013459537	0	0.016505355	0.036483147
GSM1820731_Control	0.019744316	0	0	0.043198894	0	0.084141394	0.030682665	0.007052394	0	0	0.023106654	0.018195377	0.08518117	0.371724688	0.028793388	0.224964193	0.015174569	0	0	0	0	0.048040298
GSM1820732_Control	0.007316438	0	0	0.037102209	0	0.17199135	0.099659395	0.004844372	0	0	0	0.030835991	0.117848686	0.209688882	0.033474707	0.244383471	0.004909856	0	0	0.002304089	0.004160065	0.031480552
GSM1820733_Control	0.016579235	0	0	0.049749864	0	0.056038802	0.031418327	0	0	0	0.040255865	0	0.293590758	0.330056073	0.00696315	0.145767847	0.013030789	0	0.002290138	0.013658929	0	0.000600223

GSM1820734_Control	0.012934051	0	0	0.042191152	0	0.100232753	0.026309411	0	0.001536241	0	0.026939589	0	0.178678534	0.412210249	0.021579863	0.130482254	0.011390423	0	0	0.019790087	0.005592833	0.009797064
GSM1820735_Control	0.022262545	0	0	0.047385322	0	0.112518783	0.011846767	0.021992322	0	0	0	0.021973305	0.072991654	0.339454934	0.018129853	0.228844302	0.01070524	0	0	0.034565051	0.020085569	0.037244353
GSM1820736_Control	0.027263512	0	0	0.048912879	0.018724124	0.124826664	0.054889133	0.001895728	0	0	0	0.010776119	0.105239618	0.240309233	0.010110224	0.256018759	0.020237677	0	0.009775582	0	0	0.071020748
GSM1820737_Control	0.017695133	0	0	0.036537347	0	0.03926912	0.018030773	0.040029744	0.021987429	0	0	0.026037764	0.0547253	0.291099985	0.007351322	0.377538702	0.011624774	0	0	0.029128189	0.007360542	0.021583877

GSM1820738_Control	0.029504056	0	0	0.071406303	0	0.099683125	0.027578088	0.005397719	0	0	0.018661152	0.005404739	0.108698226	0.2638048	0.009276023	0.274128933	0.008868215	0	0	0.037856013	0.003732815	0.035999792
GSM1820739_Treat	0.009866439	0	0	0.026444192	0	0.103170237	0.008027342	0	0	0	0	0.008751689	0.279299571	0.396728993	0.004688177	0.121151717	0.014534677	0	0.02157175	0	0.005765216	0
GSM1820740_Treat	0.006112216	0.007515052	0	0.024971751	0	0.08880165	0.01009965	0	0	0	0.01143919	0	0.057981324	0.423675108	0.008018351	0.296588362	0.014467183	0	0.037936182	0	0.000789359	0.021899894
GSM1820741_Treat	0.023724079	0	0	0.064229109	0.007175703	0.048683141	0.03058366	0.010699155	0	0	0	0.027096628	0.091999147	0.263474964	0.007357371	0.339535297	0.016351574	0	0.008525846	0.002140198	0.009223893	0.049200234
GSM1820742_Treat	0.00781158	0	0	0.020421976	0	0.106348926	0	0	0	0	0	0.048442686	0.229241191	0.244213583	0.003161564	0.210603636	0.005624779	0	0	0.071770044	0	0.052360035

GSM1820743_Treat	0.024757587	0	0	0.073180591	0	0.097209385	0.025201189	0	0	0	0	0.026273134	0.167143243	0.26391455	0.005990104	0.2930597	0.011322883	0	0	0.011947634	0	0
GSM1820744_Treat	0.019436613	0	0	0.034276378	0.013057999	0.071146986	0.022085431	0	0	0	0.025959385	0.000365605	0.241343754	0.363046385	0.006578731	0.164099775	0.014401187	0	0.024201771	0	0	0
GSM1820745_Treat	0	0.037323399	0	0.081443536	0	0.026149938	0.036363122	0.01563282	0	0.00741281	0	0.036146505	0.272729905	0.264020608	0.006870514	0.168914259	0.016705841	0	0.030286742	0	0	0
GSM1820746_Treat	0.01627913	0	0	0.054117551	0	0.094442612	0.01992344	0.007532241	0	0	0.001501655	0.016823165	0.043584961	0.341769913	0.01691237	0.289831538	0.013172409	0	0.017027391	0	0.002501784	0.064579839
GSM1820747_Treat	0.019627856	0	0	0.059694045	0	0.093979256	0.034988857	0.002583746	0	0	0.017970077	0.00136165	0.14643927	0.293991382	0.021789822	0.23286625	0.014323673	0	0.031368473	0	0.002796843	0.026218801

GSM1820748_Treat	0.015360157	0	0	0.019967727	0	0.017720182	0.02628539	0.020920258	0	0	0.012982323	0.012056898	0.156370125	0.431775491	0.019481187	0.202370112	0.01219018	0	0.002682838	0.002492996	0.008336015	0.03900744
GSM1820749_Treat	0.009984905	0	0	0.017788435	0	0.081870428	0	0.001586485	0	0	0	0.030003915	0.158831556	0.546259696	0.006944564	0.111466294	0.00758955	0	0.001184414	0	0.001914435	0.024575321
GSM1820750_Treat	0.018896925	0	0	0.022277908	0.003778506	0.08461099	0.029025327	0.001371105	0	0	0	0.003342703	0.070803534	0.443425803	0.008753626	0.211537967	0.018164812	0	0.034160344	0	0	0.04985045
GSM1820751_Treat	0.026065879	0	0	0.045186897	0.009078506	0.043307391	0.057464164	0.027121364	0	0.002185626	0	0.055905496	0.082083126	0.305723409	0.026812886	0.211123329	0.016194581	0	0	0.04748558	0.009801255	0.03435055
GSM1820752_Treat	0.016138172	0	0	0.040678194	0	0.149000357	0.031387695	0	0	0	0.012510114	0	0.123643868	0.331302824	0.007451953	0.193865313	0.00898808	0	4.38E-05	0.01473581	0	0.070253839

GSM1820753_Treat	0.0044677714	0	0	0.05509179	0	0.085823786	0.06149726	0.003572816	0.001982757	0	0	0.051928148	0.229971274	0.202915855	0.02083768	0.264520704	7.01E-05	0	0	0	0.001797339	0.015522799
GSM1820754_Treat	0.0017122713	0	0	0.041768905	0	0.07955494	0.033595526	0.007869627	0	0	0.008874466	0	0.068568383	0.419798282	0.009057496	0.220023192	0.015261264	0	0.022464746	0	0.004921812	0.051118649
GSM1820755_Treat	0.0015572069	0	0	0.036083239	0	0.061772415	0.003363129	0.00935278	0	0	0	0.041699477	0.053623451	0.380950058	0.001855343	0.316624314	0.013173127	0	0.030988524	0	0.001327057	0.033615018
GSM1820756_Treat	0.0011298686	0	0	0.039086751	0	0.089536589	0.017196009	0	0	0	0.029464601	0	0.162387684	0.352445598	0.005980918	0.236270564	0.00607703	0	0	0.014306398	0	0.035949173
GSM1820757_Treat	0.0009277503	0	0	0.110087201	0	0.028472707	0.060904457	0.001548903	0	0	0	0.05546474	0.356581974	0.194105047	0.005092917	0.155010503	0	0	0.003552136	0.019141351	0.000760559	0

GSM1820758_Treat	0.015675149	0	0	0.021434612	0.001547431	0.088177038	0.022892689	0	0	0	0.01329474	0.006182675	0.152640567	0.395127152	0	0.183277167	0.018504075	0.008747186	0.024719218	0	0.013065482	0.034714819
GSM1820759_Treat	0.014319377	0	0	0.014741341	0	0.146889022	0.005451559	0	0	0	0	0.020751831	0.383751635	0.287528502	0.003815056	0.072848538	0.018370069	0	0.03153307	0	0	0
GSM1820760_Treat	0.020733029	0	0	0.044202922	0.002618138	0.050179643	0.080433519	0	0	0	0.04856285	0.03697485	0.202125316	0.315101845	0.00950335	0.179141907	0.014855604	0	0.036810504	0	0.019989214	0.015751239
GSM1820761_Treat	0.010316707	0	0	0.031405169	0	0.017115704	0	0.014321198	0	0	0	0.048002668	0.105666357	0.459034715	0.006113639	0.138067522	0	0	0	0.127574756	0.007013776	0.035367793
GSM1820762_Treat	0.010634985	0.000206554	0	0.026371875	0.026383191	0.039810077	0	0	0	0	0	0.008454039	0.094418899	0.394827977	0.012782225	0.320621384	0.006727245	0	0	0.038960583	0	0.019800966

GSM1820763_Treat	0.021222954	0	0	0.047522883	0.023063302	0.025674395	0.024705098	0.005582664	0	0	0	0	0.142170811	0.392416795	0.006032293	0.243977707	0.010534042	0	0.021871609	0	0	0.035225448
GSM1820764_Treat	0.026791796	0	0	0.045456076	0	0.070087631	0.158672805	0.042634607	0	0.029400312	0	0.066233083	0.216308302	0.081406298	0.026431551	0.173969968	0.013576719	0	0	0.041538685	0	0.007492455
GSM1820765_Treat	0.034252061	0	0	0.040875144	0	0.085013646	0.025319346	0	0	0	0.021179838	0	0.128006202	0.416377404	0.007379212	0.19501761	0.010015407	0	0	0.009608086	0.004758708	0.022197335
GSM1820766_Treat	0.020702311	0	0	0.057454475	0.015220932	0.016253963	0.007910964	0.009323276	0	0	0	0.041658922	0.209195338	0.352796352	0.007348083	0.21555978	0.012298207	0	0	0.026419247	0.006701655	0.001156494
GSM1820767_Treat	0	0.00546874	0	0.046770641	0	0.068223282	0.049425475	0.016457039	0	0	0	0.032870375	0.164597816	0.22849918	0.010088894	0.266401939	0.01632935	0	0	0.046116061	0.00667164	0.042079568

GSM1820768_Treat	0.014570075	0	0	0.068325952	0	0.070913898	0.023628183	0.016139904	0	0	0	0.04079332	0.098002256	0.321244446	0.006340178	0.224510811	0.009044888	0	0	0.083145057	0.003153422	0.02018761
GSM1820769_Treat	0	0	0	0.084744426	0	0	0.044940437	0.018549531	0	0	0	0.061336044	0.111599908	0.190779539	0.023003331	0.183023065	0	0	0	0.191529002	0	0.090495711
GSM1820770_Treat	0.033068107	0	0	0.032843094	0.009083801	0.093803408	0.024383687	0	0	0	0	0	0.118741672	0.354442819	0	0.297440677	0.012546146	0	0.012961749	0	0.005011193	0.005673647
GSM1820771_Treat	0.009310778	0	0	0.040428608	0	0.053608214	0.042266101	0.003849851	0	0.003557167	0	0.024151566	0.425227301	0.160344615	0.024030291	0.195703706	0	0	0.017521799	0	0	0
GSM1820772_Treat	0	0	0	0.04089137	0.042586257	0.040596642	0.025506383	0	0	0	0.005829591	0.015957401	0.177290741	0.330440971	0.005156228	0.256044138	0.014071553	0	0.026792074	0	0	0.018836652

GSM1820773_Treat	0	0.009580118	0	0.0321491997	0.009803632	0.071546969	0.030663251	0.007681189	0	0	0.001229008	0.003974914	0.1752704666	0.4033446	0.021536617	0.177125832	0.012962308	0	0	0.023847444	0.003891652	0.015392804
GSM1820774_Treat	0.003120923	0	0	0.060311714	0	0.055209271	0.130080593	0	0	0	0	0.069608173	0.134331139	0.199443932	0.020319812	0.238034709	0	0	0	0.065589893	0.01139104	0.0125588
GSM1820775_Treat	0.031527974	0	0	0.037403088	0	0.037527124	0.012044185	0.0076857	0.008653377	0	0.011835365	0	0.111110443	0.394933914	0.007780606	0.251130141	0.011896443	0	0	0.028942165	0.010471277	0.037058198
GSM1820776_Treat	0.01511061	0	0	0.020553368	0	0.004737283	0	0.032464388	0.026395486	0	0.017700728	0	0.229116183	0.512244209	0.005296166	0.109991981	0.012553605	0	0.001181398	0	0.012654595	0
GSM1820777_Treat	0.024437991	0	0	0.098592513	0	0.011803208	0.044943237	0.070198816	0	0.000290997	0	0.047191149	0.049745933	0.342854006	0.019322719	0.195142029	0.016466321	0	0.00371189	0	0.018980804	0.056318388

GSM1820778_Treat	0	0	0	0.016978308	0	0.018384546	0.004713527	0.00116371	0	0	0	0.033188634	0.200237078	0.428831129	0.004602025	0.143158584	0	0	0	0.136699536	0.012042923	0
GSM1820779_Treat	0.024076938	0	0	0.421755143	0.036932407	0	0.054740518	0	0	0	0.020707805	0.017200734	0.412042275	0.007538993	0	0	0	0	0.005005187	0	0	0
GSM1820780_Treat	0.019233586	0	0	0.027690124	0	0.087809161	0.030260265	0	0.00276769	0	0	0.009805169	0.218920724	0.380433615	0.009270336	0.142700904	0.018612006	0	0.002133967	0.006902306	0	0.043460145
GSM1820781_Treat	0.010236773	0	0	0.02602623	0.012493029	0.12670806	0.005659084	0	0	0	0	0.050305394	0.327070572	0.215563713	0	0.142545402	0.001558896	0.013147677	0	0.054133267	0.002098517	0.012453386
GSM1820782_Treat	0.02157688	0	0	0.036138458	0.001412763	0.111215559	0.021058155	0.004144575	0	0	0	0	0.111112831	0.335883771	0.006444735	0.257394987	0.013039632	0	0.025077195	0	0.009071115	0.046429344

GSM1820783_Treat	0.022183989	0	0	0	0.009458735	0.103732095	0.011929943	0.020931222	0	0	0	0.035109218	0.274523122	0.237647136	0.00154891	0.130389458	0.011389133	0	0	0.08666033	0.025343179	0.029153531
GSM1820784_Treat	0	0	0	0.044711418	0	0.061813125	0	0.006353155	0	0.008918729	0	0.038483294	0.087014854	0.298638987	0.009330507	0.209896287	0	0	0	0.205389428	0	0.029450217
GSM1820785_Treat	0.011114323	0.008251895	0	0.05665963	0.006113383	0.081456729	0.019553768	0.001927757	0	0	0.002123034	0.022558673	0.113890701	0.370341595	0.006366166	0.255635105	0.007581984	0	0	0.008272009	0.004401749	0.023751498
GSM1820786_Treat	0.018944148	0	0	0.014265417	0.003804236	0.108649272	0.024620209	0.007129731	0	0	0.00528133	0.016667389	0.056290372	0.397491822	0.008171241	0.229397713	0.021272292	0	0	0.011986979	0.026924835	0.049103014
GSM1820787_Treat	0.016606839	0	0	0.032131808	0.010477712	0.076382331	0.018169332	0.014590345	0	0	0	0.019711484	0.10109983	0.482586614	0.00845066	0.152866009	0.020439247	0	0	0.005948537	0.003718239	0.036821014

GSM1820788_Treat	0.004107174	0	0	0.015298451	0.003870255	0.035994052	0.002114237	0.005185605	0	0	0	0.044833993	0.137548559	0.322918959	0.033481924	0.203887916	0	0	0	0.142462217	0.014826975	0.033469682
GSM1820789_Treat	0.013059468	0	0	0.026767147	0	0.075393602	0.007210621	0.008544827	0	0	0	0.019744953	0.198315792	0.422581195	0.006310021	0.172917517	0.005966241	0	0	0.030917801	0.012270816	0
GSM1820790_Treat	0.018075638	0	0	0.036779939	0	0.109272624	0.012609759	0	0	0	0.007554755	0	0.150471992	0.385100587	0.01940168	0.21286755	0.01186127	0	0	0.015721453	0.015848134	0.004434619
GSM1820791_Treat	0.02107454	0	0	0.029596014	0	0.025029211	0.023085642	0.017184735	0	0	0.026914596	0	0.090584519	0.489296609	0.007454806	0.214321212	0.020170122	0	0.009838423	0.003584545	0.012827627	0.009037397
GSM1820792_Treat	0.009729937	0	0	0.018065187	0	0.076866405	0.006500597	0	0	0	0.014999967	0	0.316569469	0.405386173	0.005520486	0.113206902	0.007617607	0	0	0.025537269	0	0

GSM1820793_Treat	0	0	0	0.150386179	0	0	0.177465482	0.000347057	0	0	0	0.097794367	0.298605885	0.056815583	0.025622775	0.063273553	0	0	0	0.039406712	0	0.090282816
GSM1820794_Treat	0.001442836	0.007958091	0	0.024885016	0	0.097246723	0.020798853	0	0	0	0.027249543	0	0.171207222	0.408758815	0.008714508	0.135593858	0	0	0	0.072120518	0.016406974	0.007617709
GSM1820795_Treat	0.014856014	0	0.019636747	0.052069632	0	0.103058987	0.044926324	0	0	0	0	0.041530442	0.234406769	0.225062183	0.021841721	0.183842595	0.009121947	0	0	0.03858954	0	0.011057098
GSM1820796_Treat	0.011502271	0	0	0.041189296	0.00689088	0.088870388	0.012852246	0	0	0	0.036750022	0	0.093696826	0.43330752	0.005992454	0.213451324	0.00749384	0	0	0.015972319	0.003780809	0.028249807
GSM1820797_Treat	0.015188659	0	0	0.034310042	0	0.105235028	0.027905565	0	0	0	0.020285875	0	0.056158756	0.438561573	0.01656063	0.220103191	0.01106833	0	0	0.022007789	0.004304697	0.028309864

GSM1820798_Treat	0	0	0	0.015077098	0.000968828	0.025295772	0	0	0	0	0	0.046276365	0.344715696	0.353588321	0	0.115093867	0	0.006595613	0	0.079166903	0.009400366	0.00382117
GSM1820799_Treat	0.016193714	0	0	0.014389904	0	0.114075585	0	0	0	0	0	0.064164467	0.233806049	0.209208369	0.003957866	0.255983679	0	0	0	0.088220366	0	0
GSM1820800_Treat	0	0	0.007249389	0	0.013002966	0	0	0.00811206	0	0.006605242	0	0.016873362	0.08105579	0.293230333	0.00186722	0.275179917	0	0	0	0.118485087	0	0.178338635
GSM1820801_Treat	0	0	0	0.057595934	0	0.012620348	0.013666886	0.004870694	0	0	0	0.042636476	0.277454334	0.263677544	0	0.269091218	0	0	0	0.024099337	0.034287229	0
GSM1820802_Treat	0.011949831	0	0	0.097322991	0	0.063208416	0.052720791	0.007182292	0	0	0	0.056644921	0.102476061	0.260828759	0.001701874	0.247461029	0.001525903	0	0	0.037100039	0.004039668	0.040527424

GSM1820803_Treat	0.021696395	0	0	0.090112063	0	0	0	0.018888099	0.007728313	0	0	0.095478457	0.226362962	0.123740775	0.002329853	0.163495254	0.024413721	0	0	0.113650139	0.079155405	0.032948564
GSM1820804_Treat	0.017578263	0	0	0.024645187	0	0.09714243	0.016710824	0.019413828	0	0	0.00969582	0.017560433	0.019642042	0.364844441	0.006277724	0.271063749	0.012024172	0	0.030522388	0	0	0.0928787
GSM1820805_Treat	0.016475756	0	0	0.022266012	0	0.099025109	0.022372431	0	0	0	0.008569125	0.003696141	0.140689875	0.451145154	0.006832363	0.139173482	0.021925156	0	0.029307997	0	0.00680263	0.031718771
GSM1820806_Treat	0.025371618	0	0	0.047840461	0	0.045270063	0.033704634	0.022229404	0	0	0	0.031018164	0.055563428	0.231980481	0.007760918	0.276314493	0.013379156	0	0	0.151830798	0.011085059	0.046651324
GSM1820807_Treat	0.012062715	0	0	0.141359547	0	0	0.065520647	0.005155616	0	0	0.007900121	0.013936635	0.077953352	0.173183911	0.017528422	0.420919108	0	0	0	0.044086069	0	0.020393858

GSM1820808_Treat	0.004360023	0	0	0.05880658	0	0.032046903	0.035949662	0.000729421	0	0	0	0.063407117	0.123490512	0.315624335	0.019558079	0.212916635	0	0	0	0.083269443	0.005656302	0.044184988
GSM1820809_Treat	0	0	0	0.055713265	0	0.123864105	0.063369718	0.010526731	0	0	0	0.031951152	0.256162291	0.247063808	0.033698508	0.145850353	0.01220365	0	0	0.01959642	0	0
GSM1820810_Treat	0.014773184	0	0	0.02495633	0.020988704	0.082504777	0.013555722	0	0	0	0	0.018595552	0.1689621	0.422150531	0.007473383	0.14231463	0.009664178	0	0	0.044874108	0.002518866	0.026667937
GSM1820811_Treat	0.000501482	0	0	0.036646104	0	5.74E-05	0.040270846	0.012012748	0	0.022476898	0	0.027150309	0.082094778	0.204007132	0.025738534	0.429735096	0	0	0	0.034385819	0.030265071	0.054657802
GSM1820812_Treat	0.001352281	0	0	0.035407084	0	0.073144753	0.018393098	0.004070417	0	0	0	0.012138768	0.234671047	0.248977045	0.020533581	0.27092546	0.001830411	0	0	0.017090185	0	0.06146587

GSM1820814_Treat	0.015114254	0	0	0.087365622	0	0	0	0.007860322	0.010412873	0	0	0.025320609	0.28359858	0.347692133	0.006317434	0.109276119	0.006148022	0	0	0.072041299	0.024359841	0.004492892
GSM1820815_Treat	0.020212223	0	0	0.048231884	0.033917615	0.058780859	0	0.005948116	0	0	0	0.047924951	0.088115918	0.244816214	0.006154498	0.316986705	0.007059439	0	0	0.080714809	0	0.041136764
GSM1820816_Treat	0.006989942	0	0.001731912	0.012224423	0.01793124	0.093923395	0.008258446	0	0	0	0.004539053	0.013278532	0.429622022	0.237082785	0	0.109323647	0.008801845	0	0	0.035099214	0.021194259	0
GSM1820817_Treat	0.011452723	0	0	0.016292102	0.032260923	0.059982974	0	0	0	0	0	0.058487564	0.373726381	0.283049086	0	0.108110911	0	0	0	0.056637337	0	0
GSM1820818_Treat	0.012144202	0	0.012962432	0.042837034	0.010854359	0.054014107	0.023926083	0.020190851	0	0	0	0	0.11328324	0.266982834	0	0.247213312	0.007977393	0	0	0.133804401	0.014949893	0.038860052

GSM1820819_Treat	0.026557168	0	0	0.057538818	0.030240095	0.030565962	0.111308222	0.006717073	0	0	0.028092429	0.012077384	0.085255058	0.163664804	0.00676632	0.337394911	0.011983278	0	0	0.056322143	0	0.035516336
GSM1820820_Treat	0.014262614	0	0	0.031491152	0	0.106028335	0	0	0	0	0	0.033412242	0.135697868	0.371290285	0.003685999	0.199624599	0.008097599	0	0	0.047076783	0	0.049332524
GSM1820821_Treat	0.008526831	0	0	0.020324614	0	0.043020081	0	0.008916212	0	0	0	0.031373397	0.278725353	0.354409729	0.004378972	0.195786578	0.006917093	0	0	0.044374717	0	0.003246424
GSM1820822_Treat	0	0.019624771	0	0.028412551	0.012093758	0.064136118	0	0.013112405	0	0	0	0.041461517	0.079450984	0.363722621	0.00578897	0.250534781	1.49E-05	0	0	0.068649681	0.007069847	0.045927103
GSM1820823_Treat	0.014309906	0	0	0.034768234	0.014615894	0.015461404	0.017121613	0	0.011832397	0	0	0	0.283238011	0.226044509	0.005394997	0.305893027	0.01469841	0	0	0.041985315	0.014636284	0

GSM1820824_Treat	0.016912721	0	0	0.03329215	0	0.014223613	0.062992564	0.016553253	0	0	0.010162809	0	0.3424248514	0.331705	0	0.1125566698	0.004858234	0	0	0.037507953	0.016749864	0
GSM1820825_Treat	0.013395176	0	0	0.005923224	0	0.008129345	0	0.017727352	0.017243234	0	0	0.060714828	0.088072758	0.330598193	0.005987255	0.13826552	0.005796875	0	0	0.187751326	0	0.120394914
GSM1820826_Treat	0.007271342	0	0	0.109946842	0	0	0.12323752	0	0	0.002223792	0	0.046702924	0.078895663	0.28810962	0.039333799	0.224302851	0	0	0	0.044795199	0.009409737	0.025770712
GSM1820827_Treat	0.021562242	0	0	0.03353987	0.017091704	0.035703783	0.034955334	0.018004319	0	0	0	0.009177858	0.091441972	0.293766173	0.024005663	0.35309064	0.017373074	0	0.012209674	0	0.007840041	0.030237653
GSM1820828_Treat	0.014043087	0	0	0.038395345	0.008602936	0.073364376	0.025706452	0	0	0	0.037743836	0	0.044566592	0.389717239	0.006805464	0.288191873	0.011292128	0	0	0.005880025	0	0.055690646

GSM1820829_Treat	0.031379534	0	0	0.057912117	0.018337493	0.084288927	0.112465888	0	0	0	0	0.039178556	0.401078497	0.048089814	0.001968103	0.116528268	0.009528317	0	0	0.079244485	0	0
GSM1820830_Treat	0.019174529	0	0	0.053189787	0.018947074	0.014861981	0.116414597	0.020102171	0	0.029626125	0	0.034915076	0.301003576	0.160730439	0.018758299	0.142271316	0.005983209	0	0	0.064021823	0	0
GSM1820831_Treat	0.010312619	0	0	0.040457107	0.000700044	0.073679258	0	0	0	0	0	0.001752973	0.084643249	0.430247026	0.010434489	0.257275568	0.002288935	0	0	0.043566798	0.000169551	0.044472383
GSM1820832_Treat	0.020490234	0	0	0.061669291	0	0.046886413	0.032199122	0.004322324	0	0	0.046607712	0	0.067701859	0.377358606	0.008439567	0.258232958	0.014777818	0	0	0.00652273	0.001552496	0.053238869
GSM1820833_Treat	0.00100788	0	0	0.030323521	0	0.054621321	0.011585103	0.005997032	0	0	0	0.014322461	0.146020618	0.361038486	0.015335877	0.289114393	0	0	0.002524583	0	0.018238816	0.049869909

GSM1820834_Treat	0.001445512	0	0	0.216154894	0	0	0.108660357	0	0	0	0	0.017360014	0.146134118	0.152180687	0.034864276	0.299540211	0.000399135	0	0	0.01584048	0.00577185	0.001648468
GSM1820835_Treat	0.014983178	0	0	0.021602484	0.010102564	0	0.021655607	0.008258521	0.010540967	0	0.021963489	0	0.165583731	0.424565331	0.005542004	0.2421748	0.009131399	0	0.0217786	0	0	0.022117324
GSM1820836_Treat	0.014259471	0	0	0.02684858	0	0.106322491	0.017879268	0	0	0	0.014922363	0	0.134668804	0.29187146	0.005066876	0.340924683	0.012405326	0	0	0.022931398	0.011899281	0
GSM1820837_Treat	0.020359022	0	0	0.030913687	0	0.0029432	0.016847026	0.033979124	0.018898422	0	0	0	0.328044162	0.356504118	0.004148745	0.128289524	0	0	0	0.052288327	0	0.006784643
GSM1820838_Treat	0.002115236	0	0	0.008922778	0.008440629	0.027561859	0	0.003862381	0	0	0	0.020068461	0.244122685	0.32650438	0.004704082	0.271867077	0	0	0	0.075421956	0.004541796	0.001866668

GSM1820839_Treat	0.015025927	0	0	0.052311812	0	0.07466292	0	0	0.010062983	0	0.014534466	0	0.060758046	0.272717153	0.01133627	0.392778644	0.00828865	0	0	0.051648515	0	0.035874615
GSM1820840_Treat	0.008101471	0	0	0.04099456	0	0.03164743	0.019151675	0.033907839	0	0	0	0	0.279926057	0.33164381	0.005081738	0.158577061	0	0	0	0.090442952	0.000525408	0
GSM1820841_Treat	0.01899287	0	0	0	0	0.049347776	0.0179038	0.014424913	0.015437978	0	0.016568799	0	0.327038532	0.329903872	0.002339036	0.15074582	0.019742155	0	0	0.037554449	0	0
GSM1820842_Treat	0.000212985	0	0	0.054421303	0	0.08535036	0.091513683	0.018940128	0	0.005773908	0	0.074943535	0.151661431	0.210004513	0.026986329	0.220096171	0	0	0	0.022920681	0.01326917	0.023905803
GSM1820843_Treat	0.014158392	0	0	0.028946771	0	0.116434355	0.019711587	0.001959298	0	0	0.012227962	0.004012904	0.088552489	0.379359965	0	0.260032467	0.010201447	0	0	0.041297226	0.004235951	0.018869185

GSM1820844_Treat	0.02512327	0	0	0.046885502	0	0.019489591	0.084517817	0.02887946	0	0	0	0.04547637	0.108178991	0.124579578	0.019817716	0.397488241	0.014529448	0	0.017221162	0.051398745	0	0.016414107
GSM1820845_Treat	0.013409463	0	0	0.028518576	0.008673745	0.096550313	0	0.004967657	0	0	0	0.007345346	0.132160399	0.462582078	0.015519771	0.178897535	0.008491392	0	0	0.017795406	0.005600475	0.019487844
GSM1820848_Treat	0.085035007	0	0	0.010644291	0.025227353	0.029551315	0.025403944	0	0.010877169	0	0.008073156	0	0.202412917	0.286720734	0.009040904	0.158396567	0.009582243	0	0.014605724	0	0.017058276	0.026002261
GSM1820850_Treat	0.012630094	0	0	0.059455485	0	0	0	0.015228353	0.064560312	0	0.029459694	0	0.147122585	0.324906148	0.015426448	0.279306856	0.00451124	0	0	0.006648115	0	0.040744669

1.6 Supplementary Table 6. Unsupervised Clustering of IPF Samples

ID	TIMP3	SPP1	ITGB3	HTRA1	Cluster

GSM1820739_Treat	4.305558	11.178187	4.974796	7.311589	Cluster1
GSM1820740_Treat	3.675668	11.531656	5.103896	8.373311	Cluster1
GSM1820741_Treat	4.35895	10.062326	5.527537	6.936551	Cluster1
GSM1820742_Treat	4.760208	14.757156	8.811351	11.54	Cluster2
GSM1820743_Treat	5.797361	9.345736	5.631621	6.500995	Cluster1
GSM1820744_Treat	4.680861	10.668188	6.4915	7.464247	Cluster1
GSM1820745_Treat	6.960201	12.518527	7.474855	7.771609	Cluster2
GSM1820746_Treat	3.838352	9.232994	4.111204	5.159636	Cluster1
GSM1820747_Treat	5.026081	9.687954	5.008874	6.542616	Cluster1
GSM1820748_Treat	6.179898	10.285898	5.371824	6.638724	Cluster1
GSM1820749_Treat	6.002985	11.042561	6.931724	7.764864	Cluster1
GSM1820750_Treat	4.639072	10.751506	5.539284	6.695979	Cluster1
GSM1820751_Treat	4.102617	7.412039	2.411983	6.90192	Cluster1
GSM1820752_Treat	4.51983	10.384326	6.397151	7.172511	Cluster1

GSM1820753_Treat	4.840554	11.160367	6.148922	7.700225	Cluster1
GSM1820754_Treat	3.994898	10.480192	5.251865	5.820156	Cluster1
GSM1820755_Treat	6.583849	13.297543	7.56863	7.393872	Cluster2
GSM1820756_Treat	5.913197	11.419785	7.688792	8.948774	Cluster2
GSM1820757_Treat	6.084988	11.692327	6.554693	7.761474	Cluster2
GSM1820758_Treat	5.371824	12.212888	7.566366	7.278724	Cluster2
GSM1820759_Treat	7.001694	11.505776	7.427491	7.37852	Cluster2
GSM1820760_Treat	4.809712	10.798855	6.179898	6.861871	Cluster1
GSM1820761_Treat	7.86409	15.918323	8.71189	10.669553	Cluster2
GSM1820762_Treat	6.860646	12.290778	7.327904	8.46058	Cluster2
GSM1820763_Treat	3.757618	9.96797	7.507231	6.677602	Cluster1
GSM1820764_Treat	4.533415	9.559725	5.48538	7.722423	Cluster1
GSM1820765_Treat	4.756672	10.648373	4.940747	8.022624	Cluster1
GSM1820766_Treat	6.272729	11.373924	5.419459	8.262538	Cluster1

GSM1820767_Treat	5.568528	10.629866	6.748527	7.579834	Cluster1
GSM1820768_Treat	4.814471	9.459858	5.839785	7.173142	Cluster1
GSM1820769_Treat	6.699892	13.467079	8.439236	9.74051	Cluster2
GSM1820770_Treat	4.86282	10.799614	7.582787	8.720684	Cluster2
GSM1820771_Treat	4.184968	7.973639	4.509711	7.147801	Cluster1
GSM1820772_Treat	6.524736	12.813691	7.339409	7.886608	Cluster2
GSM1820773_Treat	6.160599	10.873157	6.191607	8.043432	Cluster1
GSM1820774_Treat	6.121129	12.475248	5.608637	9.318164	Cluster2
GSM1820775_Treat	4.833273	9.645892	6.096908	6.520612	Cluster1
GSM1820776_Treat	2.179297	7.631657	5.078092	5.671825	Cluster1
GSM1820777_Treat	3.246132	8.754237	3.158779	6.248794	Cluster1
GSM1820778_Treat	6.889525	12.570305	8.49228	10.094647	Cluster2
GSM1820779_Treat	2.249627	3.159282	9.618948	8.140244	Cluster1
GSM1820780_Treat	6.179275	11.78491	6.173731	7.195811	Cluster1

GSM1820781_Treat	5.924231	12.535195	8.802951	9.932633	Cluster2
GSM1820782_Treat	4.361748	7.999449	5.316312	5.585456	Cluster1
GSM1820783_Treat	6.38792	13.557151	9.015168	11.022359	Cluster2
GSM1820784_Treat	5.384375	14.293992	8.456961	10.312965	Cluster2
GSM1820785_Treat	5.923533	12.180975	7.937135	9.87677	Cluster2
GSM1820786_Treat	6.510181	14.354276	7.694642	10.09402	Cluster2
GSM1820787_Treat	4.883405	9.154619	6.122412	7.700225	Cluster1
GSM1820788_Treat	5.324191	11.456079	6.714701	9.029108	Cluster2
GSM1820789_Treat	5.258424	10.446684	5.499111	7.173142	Cluster1
GSM1820790_Treat	5.183502	8.590161	3.809176	6.404074	Cluster1
GSM1820791_Treat	5.888264	9.79326	5.300953	6.876374	Cluster1
GSM1820792_Treat	8.025346	12.75206	8.642044	10.054241	Cluster2
GSM1820793_Treat	3.608478	12.244978	5.503069	10.838675	Cluster2
GSM1820794_Treat	6.179898	11.533469	7.617469	9.058918	Cluster2

GSM1820795_Treat	6.115572	12.401939	8.144442	9.458367	Cluster2
GSM1820796_Treat	4.228569	9.431037	6.979995	8.063446	Cluster1
GSM1820797_Treat	4.829084	9.296957	5.577015	6.200123	Cluster1
GSM1820798_Treat	9.479872	15.428595	9.521504	10.278772	Cluster2
GSM1820799_Treat	7.741201	13.521974	8.39351	8.486065	Cluster2
GSM1820800_Treat	6.566884	16.468942	9.635925	12.220556	Cluster2
GSM1820801_Treat	5.139942	11.554059	6.566251	9.451597	Cluster2
GSM1820802_Treat	5.300953	10.106723	5.997603	7.375585	Cluster1
GSM1820803_Treat	7.862418	13.831757	7.256721	9.450018	Cluster2
GSM1820804_Treat	4.326083	9.932135	4.696946	7.119631	Cluster1
GSM1820805_Treat	4.037449	8.971006	4.092046	7.121439	Cluster1
GSM1820806_Treat	6.298396	11.82508	6.564242	8.180425	Cluster2
GSM1820807_Treat	7.654306	14.586124	8.183693	12.508335	Cluster2
GSM1820808_Treat	4.111204	13.208697	6.726676	7.170175	Cluster2

GSM1820809_Treat	4.895103	9.621574	6.436692	6.91304	Cluster1
GSM1820810_Treat	5.115387	9.114238	4.353807	6.537324	Cluster1
GSM1820811_Treat	4.80255	9.752752	4.430113	7.168953	Cluster1
GSM1820812_Treat	5.116783	11.34456	5.905686	7.512616	Cluster1
GSM1820813_Treat	7.579175	11.837615	6.179275	7.004149	Cluster2
GSM1820814_Treat	5.862968	11.812492	6.000937	7.820021	Cluster1
GSM1820815_Treat	6.303233	11.453392	5.930543	8.308509	Cluster2
GSM1820816_Treat	4.580048	9.110536	6.329243	8.722178	Cluster1
GSM1820817_Treat	7.452308	11.701359	6.125753	9.486352	Cluster2
GSM1820818_Treat	6.299079	12.509769	4.65784	7.477137	Cluster1
GSM1820819_Treat	4.500669	7.554505	3.6796	5.363083	Cluster1
GSM1820820_Treat	5.515336	11.6237	7.506594	8.518323	Cluster2
GSM1820821_Treat	7.582787	12.921518	8.195557	8.621623	Cluster2
GSM1820822_Treat	5.554014	13.986487	6.045333	9.163356	Cluster2

GSM1820823_Treat	5.575575	11.232052	4.835465	6.869418	Cluster1
GSM1820824_Treat	6.641309	10.849407	4.110516	7.934958	Cluster1
GSM1820825_Treat	9.388717	17.127668	9.878963	13.125767	Cluster2
GSM1820826_Treat	5.079474	10.940871	5.747493	7.355358	Cluster1
GSM1820827_Treat	5.066563	8.537777	5.124019	7.530429	Cluster1
GSM1820828_Treat	5.340602	11.078377	5.076645	7.421995	Cluster1
GSM1820829_Treat	5.023986	14.036006	6.706602	10.582124	Cluster2
GSM1820830_Treat	4.386396	12.509769	8.179275	9.983947	Cluster2
GSM1820831_Treat	4.349967	11.910805	6.584479	9.778463	Cluster2
GSM1820832_Treat	4.296805	10.692856	4.609039	6.61259	Cluster1
GSM1820833_Treat	6.279094	14.153057	5.493559	9.195084	Cluster2
GSM1820834_Treat	4.952252	11.027489	5.376762	7.397255	Cluster1
GSM1820835_Treat	5.600221	11.149483	5.853805	8.327121	Cluster1
GSM1820836_Treat	7.033866	10.671507	4.471206	7.208787	Cluster1

GSM1820837_Treat	6.815619	9.89271	4.036102	7.535029	Cluster1
GSM1820838_Treat	6.601655	13.462027	6.610014	10.189296	Cluster2
GSM1820839_Treat	5.836326	15.021618	5.860094	9.816219	Cluster2
GSM1820840_Treat	6.920748	13.755657	5.597543	9.438436	Cluster2
GSM1820841_Treat	6.203612	10.487653	3.15035	7.240586	Cluster1
GSM1820842_Treat	3.618169	12.520096	4.490832	8.353001	Cluster1
GSM1820843_Treat	6.049442	12.183624	6.652991	7.565205	Cluster2
GSM1820844_Treat	7.333946	14.001489	5.848968	11.14391	Cluster2
GSM1820845_Treat	2.516516	9.092021	2.197683	4.474066	Cluster1
GSM1820846_Treat	2.543763	8.318407	2.040544	4.427927	Cluster1
GSM1820847_Treat	2.611564	9.303112	2.760703	5.855263	Cluster1
GSM1820848_Treat	14.125375	10.09402	2.549651	3.396861	Cluster1
GSM1820849_Treat	9.49274	11.827085	3.243419	7.36857	Cluster1
GSM1820850_Treat	7.535029	13.15062	4.974796	8.322232	Cluster2

1.7 Supplementary Table 7. The genes from cluster 1 and cluster 2 by WGCNA

VENTX

FCRL1

ZNF365

TNFSF14

HOXB6

SH3BP4

SULF2

MARC1

SNAI1

CDK6

MATK

NTSR1

TPCN1

CPE

ALDH1A3

MMP1

RGS1

SH3RF1

ANO5

CXCR7

BAIAP2L1

FAM20A

DPYSL3

LOC100129322

CD93

PLIN4

IL10

TESC

STON1

GALNT14

SGPP2

C9orf47

ADAM8

AATK

SH2D4B

TMEM37

RGS2

GDF15

LAMA3

SLC16A8

RASAL2

CD5L

ST6GALNAC1

SLC19A3

ANXA3

EMP1

WIF1

ARAP3

LAMB3

BMP6

TCEA3

LRG1

C1orf116

HBG1

PPP1R14C

PLTP

ATP2A3

FN1

HS3ST1

PXN

SERPINA9

CPNE5

SCEL

SLC37A3

DUOX1

A4GNT

GPR110

PRSS23

RGNEF

DLX4

OR2A9P

SLC2A5

FAM27E3

CCL3

METTL7B

MS4A6E

MMP24

CYP4F3

GPRC5A

SCIN

LPPR4

DBN1

TPPP

IER3

CLEC4G

CLEC6A

SLC7A5

KCNJ5

FNBP1L

CHST2

RNASE1

ASPRV1

AMPD3

LOC284751

NOV

TACSTD2

F13A1

NKX2-1

PLXNC1

ELF3

FAM65B

LILRB5

SERPING1

HBM

FOLR3

MYO1D

MUC1

CYP4B1

FAM3B

CTTN

FCAR

CD24

HAMP

AR

CH25H

TMC5

CCL3L3

TM4SF19

AKAP5

PLIN5

RGL1

CYTL1

FOXQ1

LOC283050

DEFA4

SLCO4A1

LOC283392

MARCKSL1

LOC100288911

SFTA2

SFN

SEPP1

DYSF

TMEM154

CGN

CHIT1

DSP

SFTA3

RAB3IL1

LOC100506328

IGFBP2

SERPINB9

AOC3

PRDM8

VNN1

FBLN5

SLC1A7

LGMN

MTSS1

IRAK2

PID1

SELL

FAM101B

ECE1

SCG5

CLIC6

PKP3

INHBB

IFITM1

CMTM2

CELA2B

SLC7A11

ID1

S1PR3

SFTPA1

GPR56

RRAD

HRK

SUSD2

IL18R1

ADORA2A

LTF

MMP25

GPR116

AFAP1L1

OVCH1

CXCL1

PLA2G7

SFTPD

ADM

MUC21

EGR3

SPON2

CCL13

KRT23

HBQ1

LCN2

CEACAM7

CSGALNACT1

PZP

CD177

DMBT1

MALL

FOSB

EFEMP1

LAMB1

AREG

STAB1

LOC440335

WFIKKN2

CELA2A

SELENBP1

CCR3

FCGBP

SIGLEC14

KCNAB1

COL22A1

UCHL1

CXCL17

PTPRF

MET

F2RL1

KANK1

G0S2

SDS

ANGPTL4

CLDN18

MMP9

ALAS2

TMEM98

KCNJ15

SLC34A2

EPCAM

CYR61

MAL2

KRT19

STEAP4

SPINK1

APOBEC3A

SFTPC

HTRA1

RGL4

MRVI1

SOD3

CXCL14

CHI3L1

CLDN3

CLDN4

MERTK

CHST15

CLC

C8B

PI3

DFNA5

TM4SF1

ITGB3

FFAR3

CDH1

VSTM1

NALCN

AWAT2

IL8

CYP1B1

CCL7

CCL8

OLIG1

PROK2

CCL2

MMP10

PTGER3

TUBB3

CEACAM6

IL1R2

HS3ST2

TPST1

MMP7

SFTPB

HPR

SPP1

S100A12

HP

HBB

DEFA3

HBA2

HBD

ZFP57

GSTT1

1.8 Supplementary Table 8. The genes from the IPF samples and control samples by WGCNA

VENTX

FCRL1

NEU4

SH3PXD2B

ZNF365

TNFSF14

HOXB6

SH3BP4

SULF2

MARC1

SNAI1

CRIP2

MATK

CALHM3

NTSR1

C2orf71

TPCN1

CPE

ALDH1A3

MMP1

EMR1

SH3RF1

ANO5

CXCR7

BAIAP2L1

FAM20A

DPYSL3

CD93

PLIN4

IL10

STON1

GALNT14

A2M

FXYD3

SGPP2

C9orf47

ADAM8

AATK

SH2D4B

SEPT4

TMEM37

RGS2

GDF15

LAMA3

SLC16A8

SLC43A1

ST6GALNAC1

NEIL2

SLC19A3

ANXA3

EMP1

WIF1

SLC25A23

ARAP3

LAMB3

BMP6

TCEA3

LRG1

C1orf116

HBG1

PPP1R14C

PLTP

HS3ST1

ENPP6

PXN

SERPINA9

FJX1

SCEL

DUOX1

CST6

A4GNT

GPR110

PRSS23

DLX4

HSPG2

SLC2A5

FAM27E3

CCL3

METTL7B

MS4A6E

MMP24

CYP4F3

GPRC5A

SCIN

LPPR4

TPPP

C10orf10

IER3

CLEC4G

CLEC6A

SLC7A5

CHST2

MPO

RNASE1

ASPRV1

AMPD3

LOC284751

SLC40A1

NOV

TACSTD2

F13A1

NKX2-1

PLXNC1

ELF3

FAM65B

LILRB5

SERPING1

HBM

FOLR3

MYO1D

MUC1

CYP4B1

FAM3B

CTTN

FCAR

CD24

HAMP

AR

C9orf30-TMEFF1

CH25H

TMC5

CCL3L3

TM4SF19

AKAP5

PLIN5

RGL1

MFGE8

CYTL1

FOXQ1

LOC283050

SLCO4A1

LOC283392

MARCKSL1

LOC100288911

SFTA2

HOXC4

SFN

SEPP1

DYSF

TMEM154

CGN

CHIT1

SFTA3

RAB3IL1

LOC100506328

AOC3

HS3ST3B1

VNN1

FBLN5

SLC1A7

LGMN

MTSS1

PID1

SELL

SCG5

CLIC6

PKP3

INHBB

CMTM2

CELA2B

SLC7A11

ID1

S1PR3

SFTPA1

GPR56

RRAD

PGA3

HRK

SUSD2

KRT1

LTF

MMP25

GPR116

AFAP1L1

OVCH1

CXCL1

KRT17

PLA2G7

SFTPD

ADM

MUC21

EGR3

LOC388780

SPON2

CCL13

KRT23

LCN2

CEACAM7

CD177

DMBT1

MALL

FOSB

EFEMP1

LAMB1

AREG

STAB1

C14orf34

WFIKKN2

CELA2A

SELENBP1

CCR3

SIGLEC14

COL22A1

UCHL1

CXCL17

PTPRF

MET

F2RL1

KANK1

G0S2

SDS

ANGPTL4

CLDN18

MMP9

ALAS2

TMEM98

KCNJ15

SLC34A2

EPCAM

CYR61

MAL2

OR8G5

KRT19

SLPI

STEAP4

L1TD1

SPINK1

APOBEC3A

SFTPC

HTRA1

RGL4

MRVI1

SOD3

CHI3L1

CLDN4

MERTK

CHST15

TMEM45B

CLC

C8B

PI3

DFNA5

TM4SF1

ITGB3

FFAR3

VSTM1

NALCN

IL8

CYP1B1

CCL7

CCL8

OLIG1

PROK2

CCL2

MMP10

TUBB3

CEACAM6

IL1R2

HS3ST2

TPST1

MMP7

SFTPB

HPR

SPP1

S100A12

HP

HBB

DEFA3

HBA2

HBD

GSTT1

1.9 Supplementary Table 9. The genes for LASSO analysis

VENTX

FCRL1

ZNF365

TNFSF14

HOXB6

SH3BP4

SULF2

MARC1

SNAI1

MATK

NTSR1

TPCN1

CPE

ALDH1A3

MMP1

SH3RF1

ANO5

CXCR7

BAIAP2L1

FAM20A

DPYSL3

CD93

PLIN4

IL10

STON1

GALNT14

SGPP2

C9orf47

ADAM8

AATK

SH2D4B

TMEM37

RGS2

GDF15

LAMA3

SLC16A8

ST6GALNAC1

SLC19A3

ANXA3

EMP1

WIF1

ARAP3

LAMB3

BMP6

TCEA3

LRG1

C1orf116

HBG1

PPP1R14C

PLTP

HS3ST1

PXN

SERPINA9

SCEL

DUOX1

A4GNT

GPR110

PRSS23

DLX4

SLC2A5

FAM27E3

CCL3

METTL7B

MS4A6E

MMP24

CYP4F3

GPRC5A

SCIN

LPPR4

TPPP

IER3

CLEC4G

CLEC6A

SLC7A5

CHST2

RNASE1

ASPRV1

AMPD3

LOC284751

NOV

TACSTD2

F13A1

NKX2-1

PLXNC1

ELF3

FAM65B

LILRB5

SERPING1

HBM

FOLR3

MYO1D

MUC1

CYP4B1

FAM3B

CTTN

FCAR

CD24

HAMP

AR

CH25H

TMC5

CCL3L3

TM4SF19

AKAP5

PLIN5

RGL1

CYTL1

FOXQ1

LOC283050

SLCO4A1

LOC283392

MARCKSL1

LOC100288911

SFTA2

SFN

SEPP1

DYSF

TMEM154

CGN

CHIT1

SFTA3

RAB3IL1

LOC100506328

AOC3

VNN1

FBLN5

SLC1A7

LGMN

MTSS1

PID1

SELL

SCG5

CLIC6

PKP3

INHBB

CMTM2

CELA2B

SLC7A11

ID1

S1PR3

SFTPA1

GPR56

RRAD

HRK

SUSD2

LTF

MMP25

GPR116

AFAP1L1

OVCH1

CXCL1

PLA2G7

SFTPD

ADM

MUC21

EGR3

SPON2

CCL13

KRT23

LCN2

CEACAM7

CD177

DMBT1

MALL

FOSB

EFEMP1

LAMB1

AREG

STAB1

WFIKKN2

CELA2A

SELENBP1

CCR3

SIGLEC14

COL22A1

UCHL1

CXCL17

PTPRF

MET

F2RL1

KANK1

G0S2

SDS

ANGPTL4

CLDN18

MMP9

ALAS2

TMEM98

KCNJ15

SLC34A2

EPCAM

CYR61

MAL2

KRT19

STEAP4

SPINK1

APOBEC3A

SFTPC

HTRA1

RGL4

MRVI1

SOD3

CHI3L1

CLDN4

MERTK

CHST15

CLC

C8B

PI3

DFNA5

TM4SF1

ITGB3

FFAR3

VSTM1

NALCN

IL8

CYP1B1

CCL7

CCL8

OLIG1

PROK2

CCL2

MMP10

TUBB3

CEACAM6

IL1R2

HS3ST2

TPST1

MMP7

SFTPB

HPR

SPP1

S100A12

HP

HBB

DEFA3

HBA2

HBD

GSTT1

1.10 Supplementary Table 10. The results of LASSO analysis

Gene	corresponding coefficient
CXCR7	-0.088106764
HS3ST1	0.089271659
MMP25	0.01381218
MRVI1	0.03539586
TM4SF1	0.058501113
TPST1	0.085395102

2 Supplementary Figure

2.1 Supplementary Figure 1. Additional analyses figures

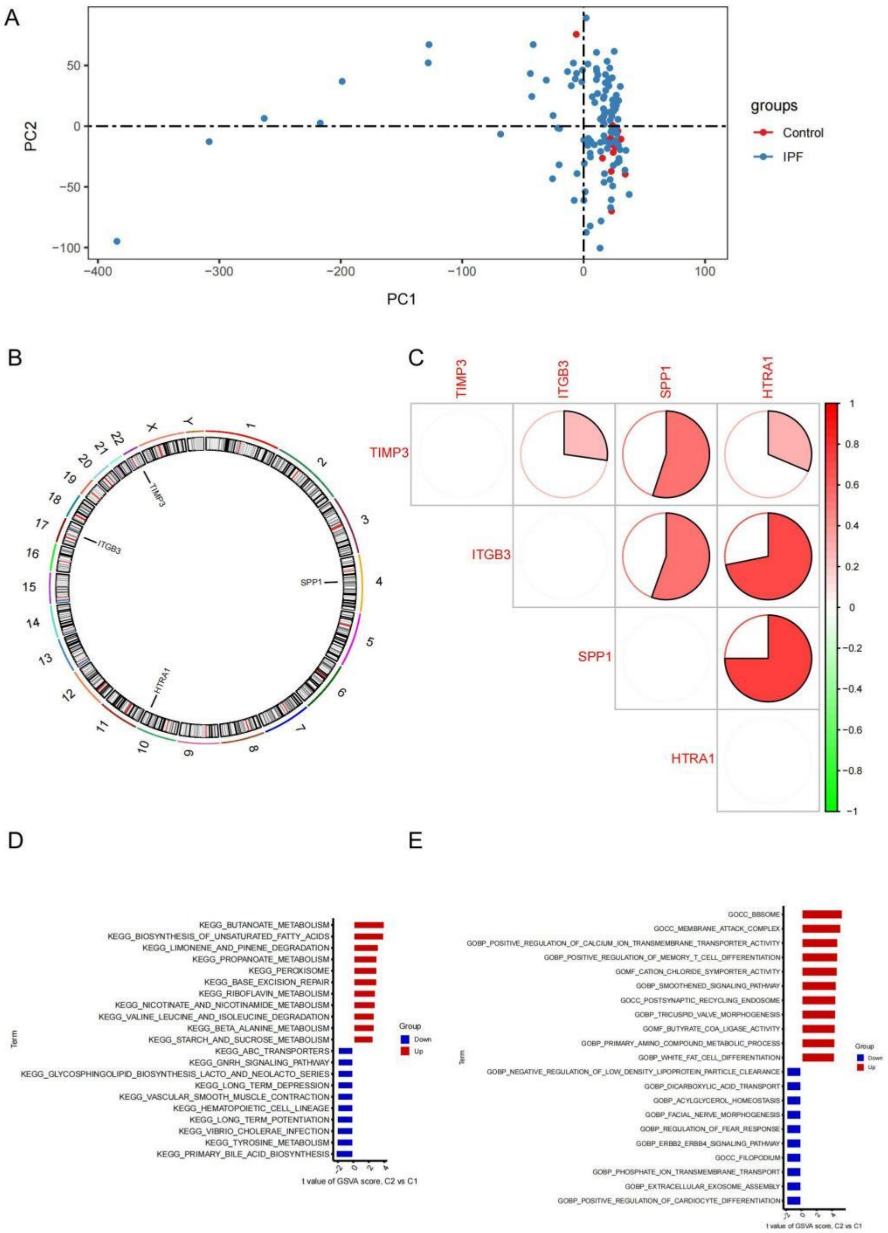
A
PC2
PC1
50
0
−50
−100
−400
−300
−200
−100
0
100
groups
Control
IPF
B
1
2
3
4
5
6
7
8
9
10
11
12
13
14
15
16
17
18
19
20
21
22
X
Y
TIMP3
ITGB3
SPP1
HTRA1
C
TIMP3
ITGB3
SPP1
HTRA1
1
0.8
0.6
0.4
0.2
0
−0.2
−0.4
−0.6
−0.8
−1
D
KEGG_BUTANOATE_METABOLISM
KEGG_BIOSYNTHESIS_OF_UNSATURATED_FATTY_ACIDS
KEGG_LIMONENE_AND_PINENE_DEGRADATION
KEGG_PROPANOATE_METABOLISM
KEGG_PEROXISOME
KEGG_BASE_EXCISION_REPAIR
KEGG_RIBOFLAVIN_METABOLISM
KEGG_NICOTINATE_AND_NICOTINAMIDE_METABOLISM
KEGG_VALINE_LEUCINE_AND_ISOLEUCINE_DEGRADATION
KEGG_BETA_ALANINE_METABOLISM
KEGG_STARCH_AND_SUCROSE_METABOLISM
KEGG_ABC_TRANSPORTERS
KEGG_GNRH_SIGNALING_PATHWAY
KEGG_GLYCOSPHINGOLIPID_BIOSYNTHESIS_LACTO_AND_NEOLACTO_SERIES
KEGG_LONG_TERM_DEPRESSION
KEGG_VASCULAR_SMOOTH_MUSCLE_CONTRACTION
KEGG_HEMATOPOIETIC_CELL_LINEAGE
KEGG_LONG_TERM_POTENTIATION
KEGG_VIBRIO_CHOLERAE_INFECTION
KEGG_TYROSINE_METABOLISM
KEGG_PRIMARY_BILE_ACID_BIOSYNTHESIS
Term
Group
Down
Up
-2
0
2
4
t value of GSVA score, C2 vs C1
E
GOCC_BBSOME
GOCC_MEMBRANE_ATTACK_COMPLEX
GOBP_POSITIVE_REGULATION_OF_CALCIUM_ION_TRANSMEMBRANE_TRANSPORTER_ACTIVITY
GOBP_POSITIVE_REGULATION_OF_MEMORY_T_CELL_DIFFERENTIATION
GOMF_CATION_CHLORIDE_SYMPORTER_ACTIVITY
GOBP_SMOOTHENED_SIGNALING_PATHWAY
GOCC_POSTSYNAPTIC_RECYCLING_ENDOSOME
GOBP_TRICUSPID_VALVE_MORPHOGENESIS
GOMF_BUTYRATE_COA_LIGASE_ACTIVITY
GOBP_PRIMARY_AMINO_COMPOUND_METABOLIC_PROCESS
GOBP_WHITE_FAT_CELL_DIFFERENTIATION
GOBP_NEGATIVE_REGULATION_OF_LOW_DENSITY_LIPOPROTEIN_PARTICLE_CLEARANCE
GOBP_DICARBOXYLIC_ACID_TRANSPORT
GOBP_ACYLGLYCEROL_HOMEOSTASIS
GOBP_FACIAL_NERVE_MORPHOGENESIS
GOBP_REGULATION_OF_FEAR_RESPONSE
GOBP_ERBB2_ERBB4_SIGNALING_PATHWAY
GOCC_FILOPODIUM
GOBP_PHOSPHATE_ION_TRANSMEMBRANE_TRANSPORT
GOBP_EXTRACELLULAR_EXOSOME_ASSEMBLY
GOBP_POSITIVE_REGULATION_OF_CARDIOCYTE_DIFFERENTIATION
Term
Group
Down
Up
-2
0
2
4
t value of GSVA score, C2 vs C1

2

Supplementary Figure 1. Additional analyses figure. (A) The PCA diagram for quality control. (B) Location of the EMT-related genes on the chromosome. (C) Correlations among 4 intersecting EMT-related genes. (D) GSVA of the KEGG database. (E) GSVA of the GO database. GO, Gene Ontology; GSVA, gene set variation analysis; PCA, principal component analysis; GG, Kyoto Encyclopedia of Genes and Genomes; EMT: epithelial–mesenchymal transition.

2.2 Supplementary Figure 2. High-risk and low-risk groups in the training and validation sets

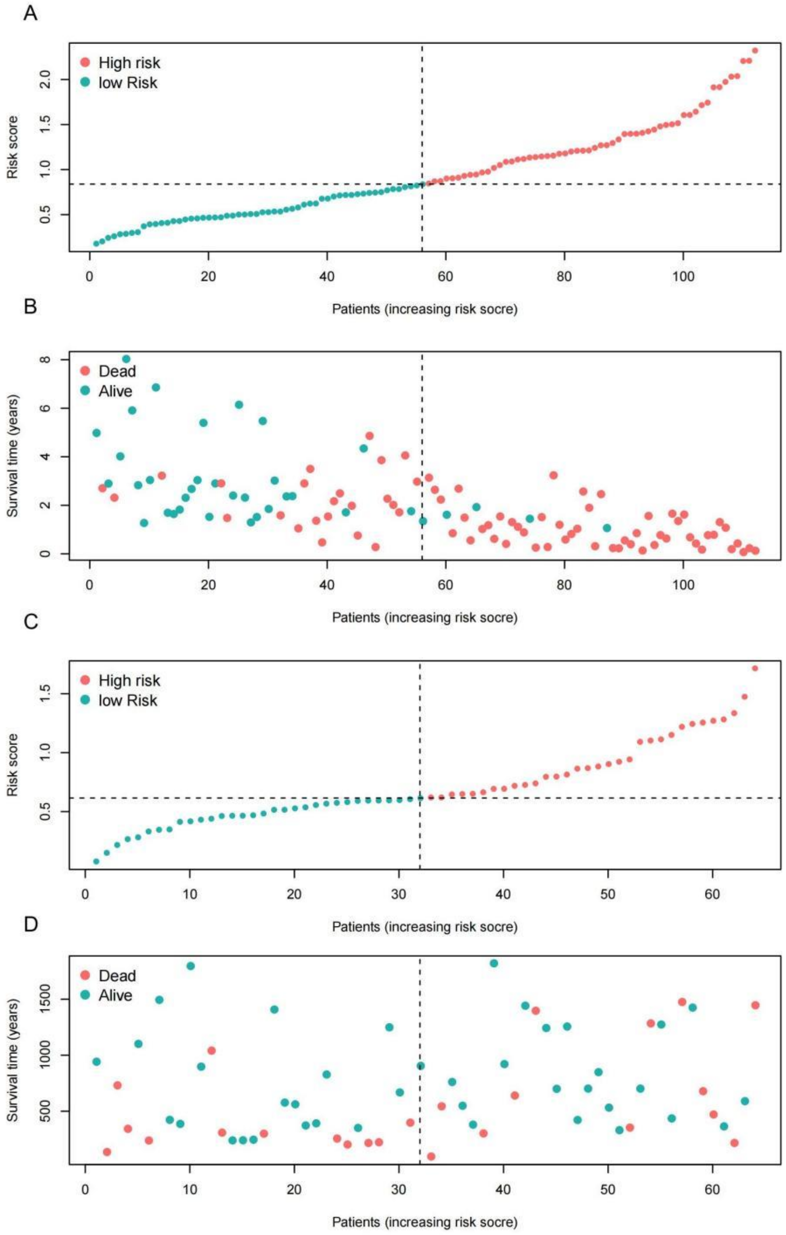
A
High risk
low Risk
Risk score
0.5
1.0
1.5
2.0
0
20
40
60
80
100
Patients (increasing risk socre)
B
Dead
Alive
Survival time (years)
0
2
4
6
8
0
20
40
60
80
100
Patients (increasing risk socre)
C
High risk
low Risk
Risk score
0.5
1.0
1.5
0
10
20
30
40
50
60
Patients (increasing risk socre)
D
Dead
Alive
Survival time (years)
500
1000
1500
0
10
20
30
40
50
60
Patients (increasing risk socre)

Supplementary Figure 2. High-risk and low-risk groups in the training and validation sets. (A) The risk curve for high-risk and low-risk groups in the training sets. (B) The survival distribution figure for high-risk and low-risk groups in the training sets. (C) The risk curve for high-risk and low-risk groups in the validation sets.(D) The survival distribution figure for high-risk and low-risk groups in the validation sets.
